# Digitalization of Access to Primary Care: Is There an Equity‐Efficiency Trade‐Off?

**DOI:** 10.1002/hec.70014

**Published:** 2025-07-17

**Authors:** Daniela Rodrigues, Noemi Kreif, Ara Darzi, Mauricio Barahona, Erik Mayer

**Affiliations:** ^1^ Department of Surgery & Cancer NIHR North West London Patient Safety Research Collaboration Institute of Global Health Innovation Imperial College London London UK; ^2^ The School of Pharmacy, Department of Pharmacy The CHOICE Institute University of Washington Seattle Washington USA; ^3^ Department of Mathematics Centre for Mathematics of Precision Healthcare Imperial College London London UK; ^4^ iCARE Digital Collaboration Space & Secure Data Environment Imperial College London & Imperial College Healthcare Trust London UK

**Keywords:** access, efficiency, equity, primary care, technology, utilization

## Abstract

In the English National Health Service, most patients can use an online platform to send a written request to the practice, in addition to calling or visiting the practice in person. However, there are concerns that the availability of an online access route to primary care can adversely impact healthcare provision for older or lower socioeconomic groups. To examine those concerns, we explore the differential timing of online platforms' implementation between 2019 and 2020 across 289 practices covering over 2.5 million patients in North West London. We find no evidence of an impact of the online access route on age and socioeconomic‐related inequity in synchronous interactions in primary care, but observe an increase in all interactions in this setting and in some cases, a small reduction (worst case, no changes) in unplanned hospital care. These findings suggest that having an online access route to primary care can improve the provision of healthcare services, at no detriment to patients from older and lower socioeconomic groups.

## 1 Introduction

Excessive delays in accessing healthcare services have become commonplace (Siciliani and Hurst 2005; Siciliani et al. 2013; OECD 2020). Some countries have turned to technology to improve access to primary care (OECD 2020). A promising initiative is the digitalization of access to primary care in the English National Health Service (NHS) which allows patients to use an online platform to send a written request to the practice at their most convenient time and location (NHS England 2019; NHS England and NHS Improvement 2020; Bakhai and Atherton 2021). Findings from G. Clarke et al. (2020) suggest a reduction in Accident and Emergency (A&E) attendances and no changes to emergency hospital admissions after the introduction of this online access route to primary care. However, there are concerns that patients using the online access route could be prioritized over other routes irrespective of clinical need (Banks et al. 2018; Carter et al. 2018; Cowie et al. 2018; Willman 2021; Parker et al. 2021; Turner et al. 2022). In particular, individuals who are older or from lower socioeconomic groups—who on average have greater clinical need—might not have the digital skills and/or resources to use the online platform (Jung and Padman 2014; Weiss et al. 2018; Rodgers et al. 2019; Zanaboni and Fagerlund 2020; Honeyman et al. 2020; Ortega et al. 2020; Darley et al. 2022). This potential “digital divide” (Hoffman et al. 2001) is a challenge also facing other sectors of the economy that are being transformed by technology.

In this study, we estimate the causal effect of having an online access route to primary care on healthcare equity and efficiency by employing a Difference‐in‐Differences (DiD) identification strategy. This strategy is based on the differential timing in the implementation of online platforms from three system providers between 2019 and 2020 across practices in North West (NW) London. In 2019, practices were encouraged to install an online platform after the commitment to the digitalization of access to primary care put forward in the *NHS Long Term Plan* (NHS England 2019). In 2020, this process was accelerated by the COVID‐19 pandemic which made alternative forms of contact such as the online channel necessary to allow practices to assess patients' clinical need before attending a face‐to‐face consultation.

We take a “horizontal” perspective on healthcare equity in which healthcare inequity arises when there is inequality (i.e., difference) in healthcare utilization among patients with the same clinical need (Wagstaff, Van Doorslaer, and Paci 1991). To assess healthcare inequity, we construct an outcome that captures inequality in healthcare utilization across patients from different age and socioeconomic groups using a concentration index. To examine healthcare efficiency, we choose the level of healthcare utilization such as the total number of patient‐provider interactions. Our analyses of the effect of the online access route to primary care on these outcome measures assume parallel trends in potential outcomes. This assumption implies that changes in important covariates such as clinical need in the case of the inequity analysis and primary care resources for the efficiency analysis have followed parallel trends over the study period. We provide supportive evidence for this assumption in Section 4.1.2. In this section, we also include a detailed examination of the potential impact of COVID‐19 in our identification strategy and show that the pandemic is likely to have impacted treated and control groups similarly and thus behave as time‐invariant confounding. To put this approach in practice, we follow the strategy developed by Callaway and Sant’Anna (2021) for the case of staggered treatment implementation. We report estimates at specific months after the implementation of the online platform (i.e., event time) to assess treatment effect heterogeneity over time, and then we combine these estimates to obtain the Average Treatment Effect on the Treated (ATT).

We find no evidence that the online access route has substantially changed the magnitude of age and socioeconomic‐related inequity in synchronous interactions in primary care or non‐urgent A&E attendances. At the same time, there has been an increase in overall patient care activity in primary care—mostly through asynchronous interactions—and in some cases, a small reduction (worst case, no changes) in unplanned hospital care activity—mostly through non‐urgent A&E attendances—for a constant change in primary care resources across practices. These results are generally robust to different versions of the intervention, proxy variables for measuring patient's socioeconomic status, concentration indices for quantifying inequality, time frames of analysis and assuming un/conditional parallel trends, which is reassuring given the potential for COVID‐19 pandemic to confound the relationship of interest. The few differences that are observed across robustness checks include a reduction in the magnitude of the patient‐provider interactions in primary care (despite the overall increase still being significant at 0.05 confidence level) and no evidence of a change in non‐urgent A&E attendances in some cases. However, these differences do not impact the main conclusion from this study which is that having online access route to primary care can lead to an increase in primary care provision, without disadvantaging patients from older or lower socioeconomic groups.

This study offers three main contributions. First, it provides the most comprehensive analysis of the effect of the digitalization of access to primary care on healthcare equity and efficiency. In contrast to the current study, most of the existing literature examining the effect of improved access to primary care—including traditional, non‐technological policy interventions (e.g., Lippi Bruni et al. 2016; Whittaker et al. 2016; Pinchbeck 2019)—do not address equity concerns or assess efficiency gains within primary care. An important exception is the work by Whittaker et al. (2019) who found that 7‐day extended access to primary care services in England benefitted younger patients the most. However, the authors also acknowledged that this outcome could be a result of previously unmet need and highlighted potential positive consequences of this initiative for other patient groups through a reduction in demand during standard hours. Furthermore, there is a vast literature in health equity (e.g., O’Donnell and Propper 1991; Van Doorslaer et al. 2000; Bago d’Uva et al. 2009) that has been important at identifying existing inequalities and inequities in health and healthcare across countries, medical specialties and patients' socioeconomic backgrounds. However, there has been less focus on examining the impact of individual policy interventions on health and healthcare equity. Our study contributes to the new paradigm in health policy evaluation in which healthcare equity is fully considered for a more complete understanding of impacts.

Second, we propose a novel approach to the assessment of equity and efficiency that takes full advantage of the DiD identification strategy. Taking the case of equity, we emphasize how changes to inequality in healthcare utilization due to the intervention can represent inequity if it is reasonable to assume that the average change in patients' clinical need over time is similar across treated and control groups. This “assumption of parallel trends in need” has been put forward by Cookson et al. (2012) when examining change in socioeconomic‐related equity in the utilization of secondary care services over time across groups with different deprivation levels. Our approach extends Cookson et al. (2012) by allowing for the estimation of the causal effect of a particular policy intervention on healthcare equity and efficiency. It is worth pointing out that our approach does not aim to estimate current levels of age or socioeconomic‐related inequity at practices. Instead, we estimate the change in the current levels of age or socioeconomic‐related inequity that is due to the intervention. The same idea applies to the assessment of efficiency. In this case, we highlight how changes to the level of healthcare utilization due to the intervention can represent efficiency improvement/loss if it is reasonable to assume that the average change in primary care resources over time is similar across treated and control groups.

Third, we also contribute to the growing literature on the impact of the digital transformation in society. Robust quantitative evidence is emerging in the education and employment sectors (e.g., Goudeau et al. 2021; Kizilcec et al. 2021). However, the inherent complexity of healthcare has led to few robust policy evaluations of technology in this context, albeit the vast public interest about its potential benefits and risks (Budd et al. 2020).

## 2 Background

**FIGURE 1:**
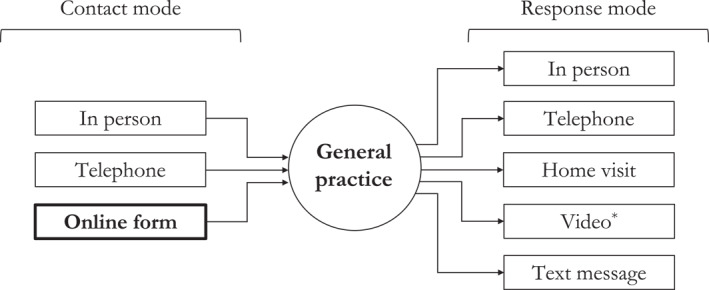
Primary care model in England, adapted from NHS England (2023). * Includes communication using video or audio only through a digital medium.

Figure 1 illustrates the primary care model in England, making the distinction between access (patient contact) and utilization (clinical response).

Currently there are three access routes for patients to contact practices, namely in person, telephone and online form (new). As an alternative to the traditional access routes, the online channel aims to tackle unmet need by making it easier for patients to contact primary healthcare professionals when necessary, rather than using A&E or foregoing care altogether which may result in (avoidable) hospitalizations in the long term. There is also the assumption that some patient queries can be resolved by messaging or calling the patient with the support of non‐medical staff, freeing up General Practitioner (GP) time to consult in person with patients with more complex needs, while allowing more patients to be treated overall. As a result, the average waiting time is expected to decrease. This expected behavior implies that the problem of excessive waiting times in primary care is driven by quantity supplied being below the efficient level, rather than excess demand due to consumer moral hazard. Despite potential benefits, the online access route to primary care could lead to healthcare inequities, particularly in synchronous primary care interactions. Patients who are younger or from higher socioeconomic groups are more likely to use the online channel (Jung and Padman 2014; Weiss et al. 2018; Rodgers et al. 2019; Zanaboni and Fagerlund 2020; Honeyman et al. 2020; Ortega et al. 2020; Darley et al. 2022). Yet, patients from older and lower socioeconomic groups have been the frequent attenders in primary care due to their higher level of clinical need. The key concern is that patients using the online channel might be offered face‐to‐face interactions with the GP that would have otherwise gone to frequent attenders.

Before 2020, only a few practices had implemented an online platform and its actual use (i.e., adoption) was very low (Edwards et al. 2017; Carter et al. 2018; Cowie et al. 2018; Eccles et al. 2019). With the publication of the *NHS Long Term Plan* (NHS England 2019)—which committed for the first time to offer all patients in England an online access route to primary care by 2023/24—more practices were encouraged to implement an online platform, but the total number of practices that did so remained low. It was only in 2020, with the COVID‐19 pandemic and the need for social distancing, when most practices in England implemented an online platform so that patients could contact them online prior to attending a face‐to‐face consultation, this way minimizing the spread of SARS‐CoV‐2 in the community (Bakhai 2020). During this time, the implementation of online platforms occurred in a “staggered” fashion, mostly driven by system providers' availability. Clinical commissioning groups—now replaced by integrated care systems—became responsible for the selection and procurement of the online platform(s) for their region from a list of approved providers compiled by NHS central organizations (NHS Digital 2023a). Online platforms were made available for free to practices, along with operational support from their clinical commissioning group.

Finally, it is worth noting that worldwide the digitalization of *access* to healthcare is still a relatively new initiative, in contrast with the digital *provision* of healthcare (also known as telemedicine).

## 3 Data

We curate a dataset for the population in NW London within the Whole Systems Integrated Care (WSIC) database in a secure data environment. WSIC contains de‐identified patient‐level data from health and social care services in NW London (North West London Integrated Care System, n.d.; Bottle et al. 2020). We enhance our dataset in WSIC with additional information about practices in NW London such as the number of online requests submitted by patients via eConsult, Klinik and askmyGP and online messages sent by practices via accuRx, and also publicly available data (see Supporting Information S1: Table S1 for more details). The final (panel) dataset is comprised of practice‐level data by calendar month from January 2018 to June 2021.

### 3.1 Sample and Population

The sample is comprised of 289 practices (see Supporting Information S1: Figure S1 for eligibility criteria). As of January 2019,^1^ the average number of patients per practice is 6937. Male and female patients are equally distributed across practices, with the elderly population (65 years or more) constituting 12% of the registered population on average. The overall mean income, employment and education decile of registered patients is the 5th, 6th and 7th, respectively, with the 10th corresponding to the least deprived decile. Regarding the workforce, there are on average 2342 and 1339 registered patients for a full‐time equivalent GP and administrative/non‐clinical staff member, respectively, across practices. For more details about the study sample, see Supporting Information S1: Table S2.

The population to whom the results can be generalized is the population in London given the key characteristics shared across the city (Dunn et al. 2022; Office for National Statistics 2022; NHS Digital 2023c).

### 3.2 Policy Intervention

The intervention under study is the online access route to primary care. We focus on implementation (availability) of this route, but also examine adoption as a robustness check.

We define implementation in terms of whether the practice installed (1) or not (0) an online platform to allow patients to contact them online. The month at which the online platform is installed at each eligible practice acts as their “time zero” (Hernán and Robins 2016; Ben‐Michael et al. 2021), with practices becoming “treated” from this month onwards. The control group consists of eligible practices that did not install an online platform up to that point in time (so‐called “not yet treated”) or ever in the study period (so‐called “never treated”).

To categorize adoption, we follow the Investment and Impact Fund (IIF) (NHS England and NHS Improvement 2021) which set a minimum activity level of five online requests per 1000 registered patients per week on average, equivalent to 22 per month for primary care networks to show a “functioning” online access route. In this study, we apply this minimum activity level to individual practices. Thus, a practice is considered to have (1) or not (0) a “functioning” online access route if it ever met (1) or not (0) the IIF threshold of 22 online patient requests per 1000 registered patients per month. In this context, time zero is the month at which eligible practices meet the IIF threshold for the first time. From this month onwards, practices are deemed “treated”. Eligible practices that did not install an online platform or have not met the IIF threshold up to that point in time constitute the control group.

**FIGURE 2:**
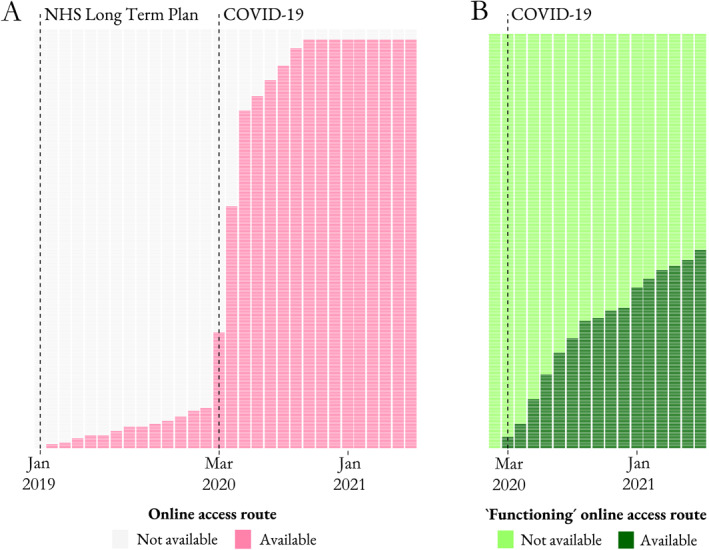
Implementation (Panel A) and adoption (Panel B) of the online access route.

From Figure 2, it is possible to identify the month at which each eligible practice started offering patients an online access route (Panel A) and first met the IIF threshold (Panel B).

### 3.3 Outcome Measures

We focus on two sets of outcome measures that correspond to the *level* and *inequality* in healthcare utilization, as per Table 1.

**TABLE 1:** Outcome measures considered in this study.

#	Measure of healthcare utilization	Setting	Level	Inequality
1	All patient‐provider interactions^a^	Primary care	Yes	No^b^
2	Asynchronous patient‐provider interactions^a^	Primary care	Yes	No^b^
3	Synchronous patient‐provider interactions^a^	Primary care	Yes	Yes
4	Synchronous patient‐GP interactions^a^	Primary care	Yes	Yes
5	Synchronous patient‐nurse interactions^a^	Primary care	Yes	Yes
6	Synchronous patient‐other‐staff^c^ interactions^a^	Primary care	Yes	Yes
7	F2F patient‐provider interactions^a^	Primary care	Yes	Yes
8	Telephone patient‐provider interactions^a^	Primary care	Yes	Yes
9	Home‐visit patient‐provider interactions^a^	Primary care	Yes	No^d^
10	Video patient‐provider interactions^a^	Primary care	Yes	No^d^
11	F2F patient‐GP interactions^a^	Primary care	Yes	Yes
12	Non‐urgent A&E attendances^a^	Secondary care	Yes	Yes
13	Potentially preventable hospital admissions^a^	Secondary care	Yes	No^d^

Abbreviations: A&E, accident & emergency department; F2F, face‐to‐face; GP, general practitioner (family doctor).

^a^ Per 1000 registered patients.

^b^ Patient‐level data not available for this variable (only practice‐level).

^c^ Other staff providing direct patient care such as pharmacists and allied health professionals.

^d^ Low overall level, with multiple practice‐months showing zero events for this variable.

We start by categorizing healthcare utilization^2^ in primary care in two groups: synchronous and asynchronous. Synchronous interactions are defined as encounters in which the healthcare professional and patient, respectively, provides and receives care in real time (e.g., face‐to‐face or telephone). Conversely, asynchronous interactions occur at different points in time (e.g., text messaging). We calculate the number of synchronous interactions every month for each registered patient according to the healthcare professional (GP, nurse or other staff providing direct patient care) and communication mode (face‐to‐face, telephone, home visit and video^3^) using encounter data which include both planned and unplanned activity. Regarding asynchronous interactions, it is not possible to differentiate between a message that was sent as part of a batch process (e.g., appointment reminders) or as a response to a specific query from the patient health record. For that reason, we use practice‐level data instead, specifically the number of “ad hoc” messages sent by practices as captured by the system provider. These “ad hoc” messages are written by healthcare professionals and include a response to a specific query from the patient, review of patient's laboratory results, among others.

In terms of secondary care services, we focus on “non‐urgent” A&E attendances as categorized by NHS Digital (2020) based on the work of Mason et al. (2017), but any visits that required a dental treatment are excluded since dentistry is out of scope. We also examine potentially preventable hospital admissions by constructing two indicators from the *NHS Outcomes Framework* (NHS Digital 2022), namely “2.3.i Unplanned hospitalization for chronic ambulatory care sensitive conditions” (except dental conditions) and “3a Emergency admissions for acute conditions that should not usually require hospital admission” (except conditions under “Influenza, pneumonia” to avoid including COVID‐19‐related cases which would be difficult to prevent).

#### 3.3.1 Level of Healthcare Utilization

Once synchronous interactions are calculated for each patient across each category, we calculate the overall level of synchronous interactions for each practice. Asynchronous interactions are already provided at practice level.

#### 3.3.2 Inequality in Healthcare Utilization

To select the most suitable concentration index for measuring inequality in healthcare utilization across the distribution of age and socioeconomic status of the patient population, we follow the guidelines developed by Erreygers and Van Ourti (2011). In particular, Erreygers and Van Ourti (2011) categorize “visits to the medical sector in a time period” as a fixed and bounded variable and recommend the use of the modified concentration index, Erreygers index (Erreygers 2009) or Wagstaff index (Wagstaff 2005). In the current context, given the challenges of setting the theoretical upper bound for healthcare utilization which can span multiple communication modes and healthcare professionals, we treat this variable as fixed but only with a lower bound (zero) and choose the generalized concentration index (Wagstaff, Paci, and Van Doorslaer, 1991; P. M. Clarke et al. 2002). Following from Erreygers and Van Ourti (2011), the generalized concentration index considered in this study is given by:

(1) $$GCI_{jt}=\frac{2}{{n_{jt}}^{2}}\sum_{i=1}^{n_{jt}}w_{\mathrm{ijt}}hc_{\mathrm{ijt}}$$

where $n_{jt}$ is the total number of patients i in a practice j at a particular month t, $hc_{\mathrm{ijt}}$ is each measure of healthcare utilization included in Table 1 for each patient at a particular practice on a given month and $w_{\mathrm{ijt}}$ is the weight for each patient at a particular practice on a given month which is based on their position/rank across age and socioeconomic groups, as per $rank_{\mathrm{ijt}}-\left[\left(n_{jt}+1\right)/2\right]$. We use income deprivation levels of the patient's residential area as proxy for the patient's socioeconomic status. Specifically, we use the income ranking from the Index of Multiple Deprivation, with 1 and 32,844 corresponding to the most and least deprived areas in England, respectively (GOV.UK 2019). We also consider employment and education deprivation levels as alternative measures of the patient's socioeconomic status. As a result, $GCI_{jt}$ is estimated 32 times (8 outcome measures times 4 ranking variables) for each practice and month since January 2018 up to June 2021.

As a robustness check, we use the Erreygers index while setting the upper bound of each measure of healthcare utilization to the empirical maximum at each practice on each month. Following from Erreygers and Van Ourti (2011), the Erreygers index considered in this study is given by:

(2) $$E_{jt}=\frac{8}{{n_{jt}}^{2}\left(u_{hc_{jt}}-l_{hc_{jt}}\right)}\sum_{i=1}^{n_{jt}}w_{\mathrm{ijt}}hc_{\mathrm{ijt}}$$

(3) $$=\frac{8}{{n_{jt}}^{2}u_{hc_{jt}}}\sum_{i=1}^{n_{jt}}w_{\mathrm{ijt}}hc_{\mathrm{ijt}}$$

where the same definition of variables apply as in Equation (1), with the additional $u_{hc_{jt}}$ and $l_{hc_{jt}}$ representing, respectively, the upper and lower bounds of each measure of healthcare utilization at each practice on each month. Given that the lower bound is zero, we obtain Equation (3). Similarly, for each practice‐month set, $E_{jt}$ is estimated 32 times, with each estimation corresponding to different outcome and ranking variables.

For both indices, negative (positive) values suggest healthcare utilization is concentrated among the youngest (oldest) or those from the most (least) income, employment or education‐deprived areas. Zero represents equality, that is, patients from different age groups, or income, employment or education‐deprived areas share the same healthcare utilization patterns (Erreygers and Van Ourti 2011; O’Donnell et al. 2016). The current analysis focuses on inequalities within each practice. Finally, we use the conindex package (O’Donnell et al. 2016) in Stata (version 15.1) to estimate all indices.

## 4 Methods

### 4.1 Identification

We employ a DiD identification strategy to take advantage of the differential timing of implementation of online platforms in the NHS. The causal estimand of interest is the ATT which is given by:

(4) $$ATT=E\left[Y_{t}\left(1\right)-Y_{t}\left(0\right)|A_{t}=1\right]$$

where $Y_{t}\left(1\right)$ and $Y_{t}\left(0\right)$ are the potential outcomes for when the online access route is available and remains unavailable, respectively, at a particular point in time t among practices for which this route is available $\left(A_{t}=1\right)$. This expectation is taken over all treated practices and time periods. Outcomes include the overall level and age and socioeconomic‐related inequality in healthcare utilization (see Table 1 for all outcome measures).

To tackle this problem, we follow the approach by Callaway and Sant’Anna (2021) which generalizes the simple case of two groups and two time periods (2 × 2) to cases where multiple groups are treated at different points in time (current case). Callaway and Sant’Anna (2021) avoids what Borusyak and Jaravel (2016) call “forbidden comparisons”, that is, when already‐treated units act as the control group for units that are just being treated for the first time.^4^ Specifically, we start by focusing on the following “auxiliary” causal estimand:

(5) $$\phi_{t,G_{t0}}=E\left[Y_{t}\left(1\right)-Y_{t}\left(0\right)|G_{t0}=1\right]$$

where the difference in potential outcomes is considered among practices for which the online access route has become available *for the first time* $\left(G_{t0}=1\right)$. This estimand is specific for every group of practices that installed the online platform at the same time (time zero, t0, depicted in Figure 2) and a particular time period which resembles the simple 2 × 2 case. The key idea underlying the approach by Callaway and Sant’Anna (2021) is to split the main dataset with all groups and time periods into multiple datasets, each for a different group‐time case. Then the approach is to address identification and estimation for each group‐time case and at the end aggregate all group‐time cases to obtain the causal estimand of interest (Equation 4). With that in mind, the next step is to convert the auxiliary causal estimand (Equation 5) into the following statistical estimand by using not‐yet‐treated units as the control group:

(6) $$\phi_{t,G_{t0}}=E\left(Y_{t}-Y_{t0-1}|G_{t0}=1\right)-E\left(Y_{t}-Y_{t0-1}|A_{t}=0\right)$$

In practice, this result means that if the identification assumptions are plausible (more on this below), the causal estimand is identified as the overall difference in the outcome trend between the group that went live at the same time $\left(G_{t0}=1\right)$ and the not‐yet‐treated $\left(A_{t}=0\right)$ group. In this study, the not‐yet‐treated group also includes never‐treated units. To obtain this result, we make three key identification assumptions, namely the existence of parallel trends in potential outcomes between treated and control groups, no anticipation and no interference (Lechner 2011; Roth et al. 2023). No anticipation implies no effects of the online platform prior to implementation, which seems reasonable given that ultimately the decision to install the online platform is made by the practice. No interference means that the fact that some practices install an online platform does not influence the overall level and inequality in healthcare utilization at the other practices, which is also plausible since patients in the NHS can only use care services at the practice they are registered with and most patients choose their practice based on proximity to their residence.

#### 4.1.1 Parallel Trends Assumption

Throughout our study we assume that the overall level and inequality in healthcare utilization for patients at practices that installed the online platform would have evolved in a similar trajectory as observed for patients at practices that did not install the platform in the absence of the platform. To examine the plausibility of this assumption, we start by estimating the average treatment effects across the pre‐intervention time periods, using our main estimation approach outlined in Section 4.2. The estimates lie around zero. However, due to the limitations of this type of “pre‐trends” test (Bilinski and Hatfield 2020; Roth 2022), we further examine the role of covariates underpinning the parallel trends assumption.

Treated and control groups do not necessarily need to have a similar covariate distribution as it is generally the case in a randomized controlled trial, since DiD can handle time‐invariant confounding. However, if groups do show a different covariate composition and these covariates are expected to impact both the treatment and outcome trend, this difference needs to remain constant over time and covariates cannot have different effects on the outcome over time (Abadie 2005; Zeldow and Hatfield 2021). With that in mind, we first examine how treated and control groups compare in terms of observable characteristics, giving special attention to variables affecting both the treatment and outcome trend. Then, we assess how likely it is for these characteristics to have evolved similarly over time across treated and control groups and finally, discuss whether their effect on the outcome is likely to be time invariant.

**FIGURE 3:**
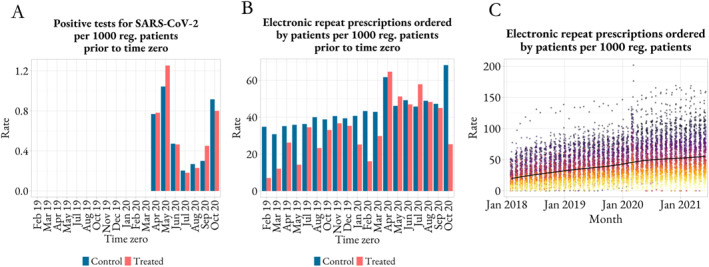
COVID‐19 testing and patient‐facing technology uptake across treated and control groups. Panel A shows pre‐treatment levels of COVID‐19 cases and Panel B, patient‐led repeat‐prescription orders across treated and control groups. Panel C depicts monthly average levels of patient‐led repeat‐prescription orders across practices. In panel C, each dot is a practice; practices are colored according to their position in the covariate distribution as of June 2021, from yellow (practices with lowest values) to dark gray (practices with highest values). Reg. indicates registered.

Supporting Information S1: Figures S2 and S3 show that treated and control groups at each time point exhibit a similar composition across many covariates and outcomes prior to the implementation of the online access route. There is a greater similarity across treated and control groups from March 2020, as if the pandemic introduced some randomness to the treatment assignment. Among all covariates, it is worth noting the close similarity in pre‐treatment levels of COVID‐19 cases across treated and control groups as shown in Panel A of Figure 3.

Yet, there are differences across treated and control groups in some cases. For example, treated and control groups display noticeable differences in terms of patient‐led repeat‐prescription orders, particularly before March 2020 as shown in Panel B of Figure 3. Nevertheless, in Panel C we can see that practices that ranked at the bottom (in yellow) in June 2021 for this covariate (i.e., with the lowest levels of patient‐led repeat‐prescription orders) are also at the bottom in the remaining months and the same is observed for practices at the top (in dark gray). The ranking is stable over time which means that practices do show differences between them but these differences are maintained over time. This finding is generally observed for the remaining characteristics of the sample as per Supporting Information S1: Figure S4, which means treated and control groups are likely to have remained constant over time.

The fact that the composition of treated and control groups is generally similar gives reassurance when it comes to the possibility of having covariates with different effects over time. In addition, practices in this study have been in operation for 40 years on average, which means that any core characteristics might have reached an equilibrium by now. An exception is anything related with patient‐facing digital technology which has increased in popularity since the pandemic, but for which the effects are still unknown.

#### 4.1.2 Parallel Trends in Clinical Need and Healthcare Resources

Our approach to examine healthcare equity is centered around the assumption of a similar average change in clinical need over time for treated and control groups. This “assumption of parallel trends in need” was also considered by Cookson et al. (2012) when studying change in socioeconomic‐related equity in the utilization of secondary care services over time across groups with different deprivation levels. Here, we extend this assumption to the study of the impact of a particular policy intervention on healthcare equity and efficiency.

**FIGURE 4:**
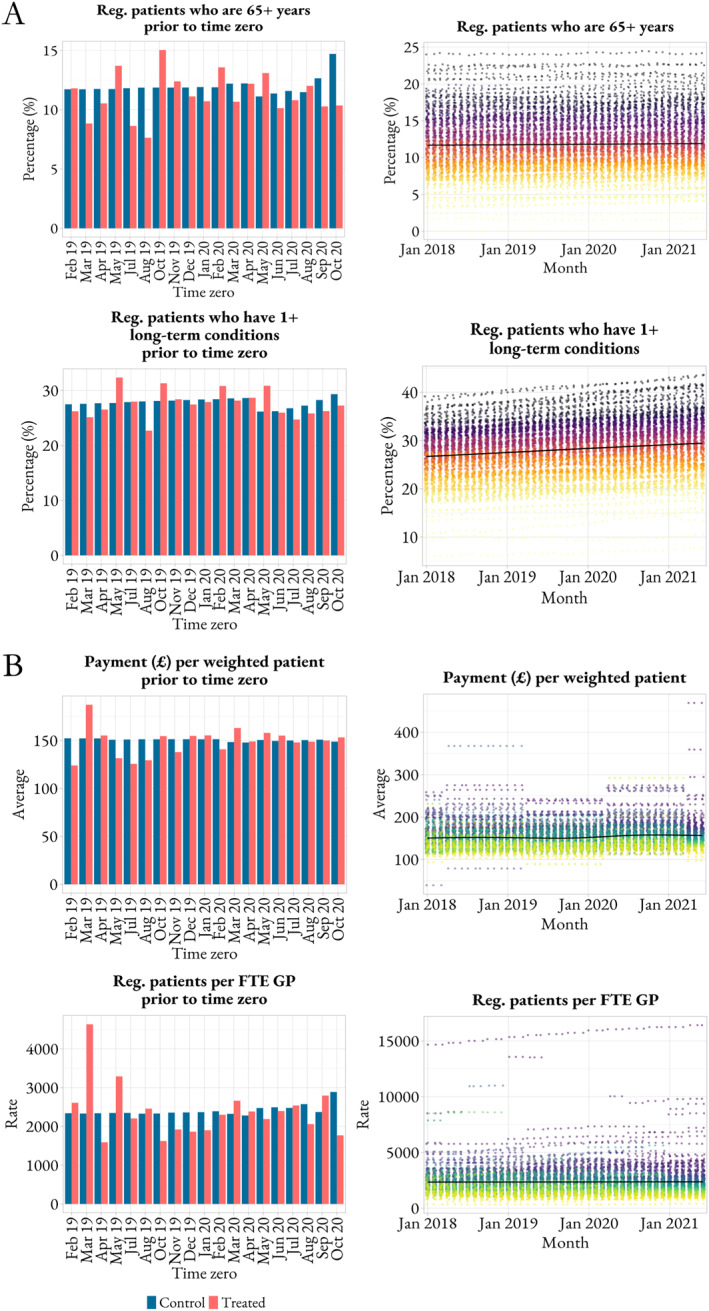
Patients' clinical need and healthcare resources across treated and control groups. Panel A shows pre‐treatment levels across treated and control groups (left‐hand side) and monthly average levels across practices (right‐hand side) for age and comorbidity profile of registered patients and Panel B, payment received and patient‐per‐general practitioner ratio. On the right‐hand side of both panels, each dot is a practice; practices are colored according to their position in each covariate distribution as of June 2021, from yellow (practices with lowest values) to dark gray/purple (practices with highest values). F2F, indicates face‐to‐face; GP, general practitioner; reg., registered.

Traditionally, patient's age and comorbidity profile are used as proxy variables for patient's clinical need. In the context of the COVID‐19 pandemic, COVID‐19 cases are naturally important to take into account as well. As shown in Panel A of Figure 3, treated and control practices have a registered patient population with similar levels of COVID‐19 cases prior to the implementation of the online access route. In addition, Panel A of Figure 4 shows that treated and control practices are responsible for a similar percentage of patients with 65 years or more and with one or more long‐term conditions prior to the intervention. It also indicates that practices treat a similar percentage of patients with 65 years or more and with one or more long‐term conditions over time. All these findings support the assumption that the average change in clinical need over time is similar across treated and control practices. Therefore, any changes to inequality due to the online access route can represent inequity.

We propose a similar approach to assess healthcare efficiency. In this context, the assumption is of a similar average change in primary care resources over time for treated and control groups. Panel B of Figure 4 shows that treated and control practices receive a similar payment and have a similar number of registered patients per full‐time equivalent GP (and non‐clinical staff as per Supporting Information S1: Figure S2) prior to the implementation of the online access route. It also suggests that the overall levels of payment to practices and patient‐staff ratio are relatively stable over time across practices. All these findings support the assumption that the average change in primary care resources over time is similar across treated and control practices. Therefore, an increase in the overall level of primary care utilization and/or reduction in non‐urgent hospital activity due to the online access route can represent efficiency improvement.

#### 4.1.3 Robustness Checks

As robustness checks for potential violations of the parallel trends assumption, we consider two additional scenarios. First, we split the main dataset into two datasets, one covering the period up to 2019 (before COVID‐19) and the other from 2020 onwards and run the analysis separately for each period. Second, we assume *conditional* parallel trends,^5^ that is, parallel trends for practices with similar characteristics prior to treatment initiation. These characteristics include (1) the number of positive tests for SARS‐CoV‐2 per 1000 registered patients, (2) percentage of registered patients who have one or more long‐term conditions, (3) number of registered patients per full‐time equivalent GP and (4) administrative/non‐clinical, (5) number of electronic repeat prescriptions ordered by patients per 1000 registered patients and (6) percentage of qualified and permanent GPs who are 55 years or more. In this case, we consider the following identification solution (Heckman et al. 1997; Heckman et al. 1998; Roth et al. 2023):

(7) $$\phi_{t,G_{t0}}^{\mathrm{condPT}}=E\left[E\left(Y_{t}-Y_{t0-1}|G_{t0}=1,X_{t0-1}\right)-E\left(Y_{t}-Y_{t0-1}|A_{t}=0,X_{t0-1}\right)|G_{t0}=1\right]$$

which is a version of Equation (6) that is conditioned on a vector X of the aforementioned six covariates measured at the time period immediately before the treated group being exposed to treatment for the first time (t0−1), and then averaged across practices that implement the online access route at the same time.

### 4.2 Estimation and Inference

To estimate the statistical estimand (Equation 6) for each group‐time case, we use sample outcome means to estimate population expectations (Roth et al. 2023) as per the below:

(8) $${\hat{\phi }}_{t,G_{t0}}=\left({\bar{Y}}_{t,G_{t0}=1}-{\bar{Y}}_{t0-1,G_{t0}=1}\right)-\left({\bar{Y}}_{t,A_{t}=0}-{\bar{Y}}_{t0-1,A_{t}=0}\right)$$

where ${\bar{Y}}_{t,G_{t0}=1}$ and ${\bar{Y}}_{t0-1,G_{t0}=1}$ are the sample outcome means at a specific time and before treatment initiation, respectively, for practices that installed the online platform at the same time; ${\bar{Y}}_{t,A_{t}=0}$ and ${\bar{Y}}_{t0-1,A_{t}=0}$ are the equivalent quantities for practices that have not yet installed the platform. This analysis implies unconditional parallel trends.

When we assume conditional parallel trends as robustness check, we start by simplifying Equation (7) using the law of iterated expectations (Heckman et al. 1997; Heckman et al. 1998; Roth et al. 2023) as per Equation (9):

(9) $$\phi_{t,G_{t0}}^{\mathrm{condPT}}=E\left(Y_{t}-Y_{t0-1}|G_{t0}=1\right)-E\left[E\left(Y_{t}-Y_{t0-1}|A_{t}=0,X_{t0-1}\right)|G_{t0}=1\right]$$

(10) $$=\left({\bar{Y}}_{t,G_{t0}=1}-{\bar{Y}}_{t0-1,G_{t0}=1}\right)-E\left[\hat{E}\left(Y_{t}-Y_{t0-1}|A_{t}=0,X_{t0-1}\right)|G_{t0}=1\right]$$

While it is straightforward to estimate $E\left(Y_{t}-Y_{t0-1}|G_{t0}=1\right)$ by replacing the population expectations for treated practices with their sample outcome means, it is more challenging to estimate $E\left(Y_{t}-Y_{t0-1}|A_{t}=0,X_{t0-1}\right)$. In this case, we follow the strategy initially developed by Heckman et al. (1997); Heckman et al. (1998) by regressing the outcome difference on the aforementioned six covariates among not‐yet‐treated practices via ordinary least squares. Then we apply this model on the distribution of covariates from treated practices to obtain predicted values. At the end, we average estimates across all treated practices.

After computing the statistical estimand for all group‐time cases, we average all estimates first at specific months after treatment initiation (so‐called “event time”) to assess treatment effect heterogeneity since implementation and then across event times to obtain the main estimate of the causal estimand of interest (Equation 4) which encompasses all groups and times. We also assess potential treatment effect heterogeneity according to several practice's characteristics such as size of registered patient population, internet access and patients' experience of the practice by following the strategy highlighted in A. Baker et al. (2025). Additionally, and as robustness check, we run a similar analysis but with a focus on the characteristics of the registered patient population as an alternative approach to using a concentration index for equity assessment. Specifically, we run two separate analyses for each characteristic of interest, each time splitting the sample into two subsamples, one subsample including practices for which the characteristic in question, for example, size of registered patient population is equal or above the median of the main sample and one subsample including practices for which the characteristic in question is below the median of the main sample. Our decision was to create two groups/subsamples rather than consider a more granular grouping in order to maximize sample size to allow for such analyses. For inference, given the large number of clusters in total (289 practices), we use the multiplier bootstrap procedure based on 1000 repetitions to obtain simultaneous confidence intervals of each estimate. This strategy avoids issues that might arise from using the same practices in the estimation process of different group‐time cases. Finally, we run the analysis at the practice level and weight practices according to their number of registered patients. We execute all these steps in R (version 4.1.0) using the did package (Callaway and Sant’Anna 2022).

## 5 Results

### 5.1 Descriptive Analysis

#### 5.1.1 Level of Healthcare Utilization

**FIGURE 5:**
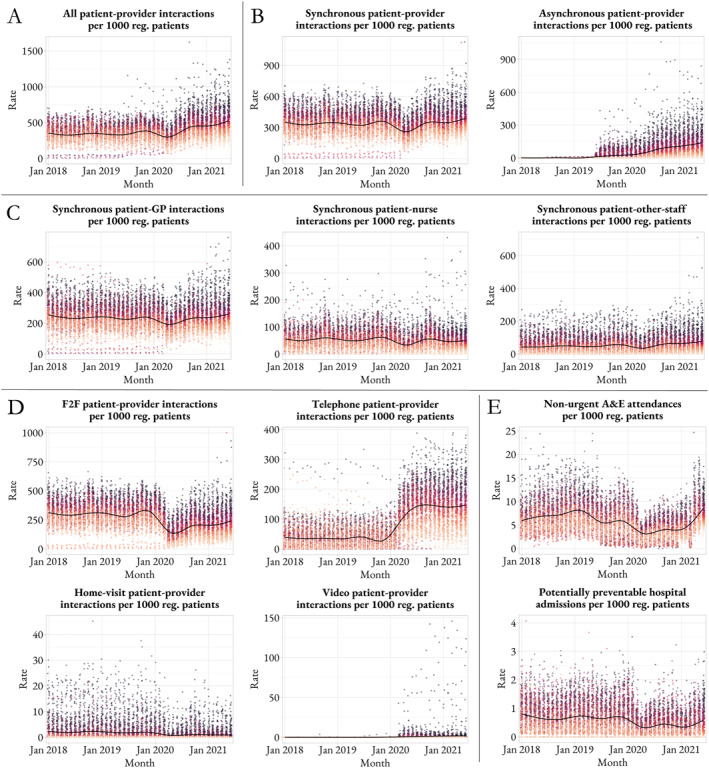
The level of patient‐provider interactions across primary and secondary care settings. Panel A depicts the number of interactions in primary care overall, Panel B, by type, Panel C, by healthcare professional and panel D, by communication mode. Panel E shows unplanned hospital care activity. The time period is from January 2018 to June 2021. Each dot is a practice; practices are colored according to their position in each outcome distribution as of June 2021, from light orange (practices with lowest values) to dark gray (practices with highest values). A&E indicates accident & emergency department; F2F, face‐to‐face; GP, indicates general practitioner; reg., registered.

Figure 5 shows the overall level of healthcare utilization across primary and secondary care services between January 2018 and June 2021. There is wide variation in the levels of healthcare utilization across practices, but these differences are stable over time in most cases. Within primary care, there was an overall reduction in synchronous interactions between April and May 2020, which occurred alongside the rise of asynchronous communication. Since June 2020, the number of synchronous interactions has returned to historical levels. Overall, there has been an increase in all interactions. While the GP and nurse are still responsible for most interactions in primary care, there has been an increase in activity by other clinical staff, such as pharmacists and allied health professionals. While the telephone acted as a substitute for face‐to‐face interactions during the first months of the pandemic, the number of face‐to‐face interactions have since increased and the number of telephone interactions seem to have hit a new plateau of more than 3 times the pre‐pandemic level. Finally, home visits and video interactions are much less frequent than those in person or by telephone, and the same is observed for non‐urgent A&E attendances and potentially preventable hospital admissions.

#### 5.1.2 Inequality in Healthcare Utilization

**FIGURE 6:**
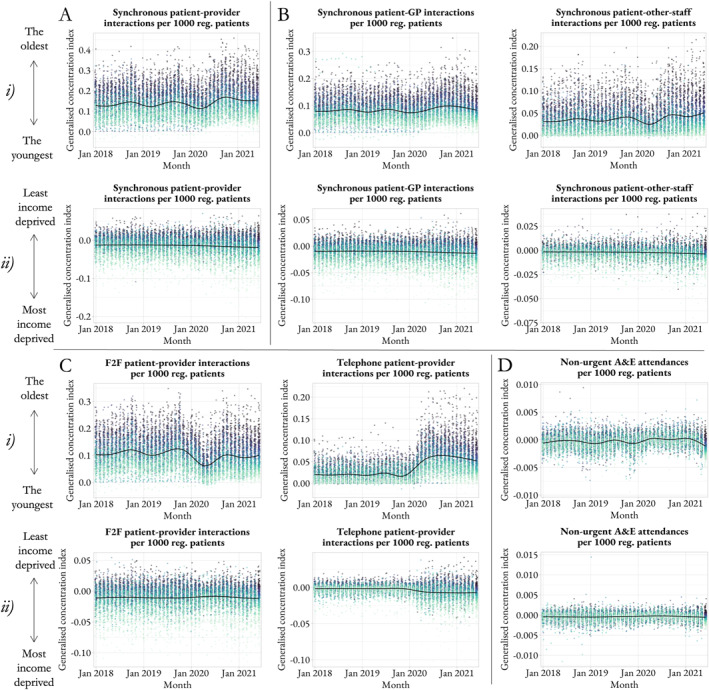
Inequality in patient‐provider interactions across primary and secondary care settings. Panels (i) and (ii) depict age and socioeconomic‐related inequality in healthcare utilization, respectively, based on the generalized concentration index. Panel A shows inequality in synchronous interactions in primary care overall, Panel B, by healthcare professional and Panel C, by communication mode. Panel D shows inequality in non‐urgent non‐urgent A&E attendances. The time period is from January 2018 to June 2021. Socioeconomic status is based on income‐related deprivation levels. Each dot is a practice; practices are colored according to their position in each outcome distribution as of June 2021, from light green (practices with lowest values) to dark gray (practices with highest values). GP, indicates general practitioner; reg., registered.

Figure 6 displays age and socioeconomic‐related inequality in healthcare utilization based on the generalized concentration index across primary and secondary care services between January 2018 and June 2021. In general, practices exhibit a constant difference over time. Positive average values in Panel (i) suggest that synchronous interactions in primary care are concentrated among the oldest patients. Between April and May 2020, there was a decrease in age‐related inequality in synchronous interactions; likely to be a reflection of the change in clinical need around that time, with more patients from younger groups, potentially affected by COVID‐19 requiring primary care. Nevertheless, age‐related inequality has since increased, that is, synchronous interactions in primary care have been further concentrated among the oldest patients. This increase has been mostly driven by an increase in interactions with other clinical staff and by telephone. Negative average values in Panel (ii) suggest that synchronous interactions in primary care are concentrated among patients living in the most income‐deprived areas. Yet, socioeconomic‐related inequality is less evident than age‐related inequality. Furthermore, the inequality index for non‐urgent A&E attendances is very close to zero across all time periods, suggesting that patients from different age groups and income‐deprived areas have similar utilization patterns of A&E due to a non‐urgent condition. Finally, we observe similar findings when using the Erreygers index as inequality measure as per Supporting Information S1: Figure S5.

### 5.2 Causal Analysis

**FIGURE 7:**
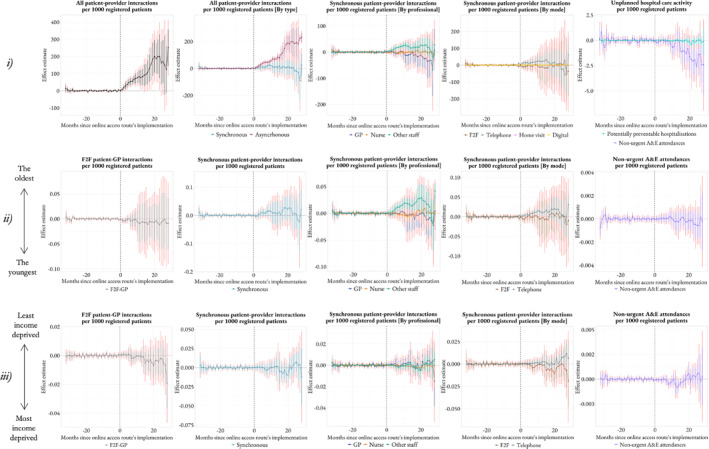
Estimates of the average treatment effect on the treated at different months since implementation of the online access route to primary care from the main analysis. Panels (i), (ii) and (iii) show, respectively, the level, age and socioeconomic‐related inequality in healthcare utilization based on the generalized concentration index. Confidence intervals that account for potential issues arising from multiple testing are shown in red.

Figure 7 shows the ATT estimates of the online access route at different months since implementation. Table 2 includes all ATT estimates averaged across all time periods.

**TABLE 2:** Estimates of the average effect on the treated of the online access route to primary care on the pre‐defined set of outcomes.

Outcome	(A)	(B)	(C)	(D)	(E)	(F)	(G)	(H)
	Main	2018–19	2020–21	Cond. PT	Adoption	Employmnt	Education	Erreygers
All patient‐provider interactions								
Level^a^	107.227*	68.139*	54.954*	56.715*	60.941*	—	—	—
Asynchronous patient‐provider interactions								
Level^a^	104.704*	51.282*	42.830*	37.351*	60.745*	—	—	—
Synchronous patient‐provider interactions								
Level^a^	3.652	17.152	12.132	19.228	0.884	—	—	—
Age‐related inequality	0.009	0.007	0.008	0.027	−0.006	—	—	0.001
Socioeconomic‐related inequality	−0.001	−0.001	0.000	0.004	0.000	0.003*	0.001	0.000
Synchronous patient‐GP interactions								
Level^a^	−11.304	4.624	−3.344	2.840	1.357	—	—	—
Age‐related inequality	−0.004	−0.002	−0.001	0.011	−0.005	—	—	0.000
Socioeconomic‐related inequality	0.000	0.001	0.001	0.005	−0.001	0.003*	0.000	0.000
Synchronous patient‐nurse interactions								
Level^a^	−0.539	−1.072	−0.439	3.094	−1.403	—	—	—
Age‐related inequality	0.000	−0.002	−0.002	0.000	−0.003	—	—	0.000
Socioeconomic‐related inequality	−0.001	−0.001	0.000	0.001	0.000	0.000	0.002*	0.000
Synchronous patient‐other‐staff^b^ interactions								
Level^a^	15.496*	13.601	15.915*	13.293	0.930	—	—	—
Age‐related inequality	0.014*	0.014*	0.010*	−0.004	0.001	—	—	0.002*
Socioeconomic‐related inequality	0.000	0.000	0.000	0.001	0.001	0.000	0.000	0.000
F2F Patient‐provider interactions								
Level^a^	−6.364	35.280	−7.566	1.404	9.750	—	—	—
Age‐related inequality	−0.001	0.011	−0.002	0.018	−0.002	—	—	0.000
Socioeconomic‐related inequality	−0.004	−0.001	−0.001	−0.001	0.000	0.001	−0.001	0.000
Telephone patient‐provider interactions								
Level^a^	9.832	−18.421	19.122	17.005*	−7.824	—	—	—
Age‐related inequality	0.010	−0.004	0.009*	0.007	−0.003	—	—	0.002
Socioeconomic‐related inequality	0.002	0.000	0.002	0.005	−0.001	0.002	0.002	0.000
Home‐visit patient‐provider interactions								
Level^a^	−0.285	0.291	0.094	0.432	0.056	—	—	—
Video patient‐provider interactions								
Level^a^	0.469*	0.001	0.482*	0.387	−1.098*	—	—	—
F2F Patient‐GP interactions								
Level^a^	−9.314	21.916*	−12.887	−5.721	10.182	—	—	—
Age‐related inequality	−0.005	0.002	−0.004	0.010	0.000	—	—	−0.001
Socioeconomic‐related inequality	−0.003	0.001	−0.001	0.000	−0.001	0.001	−0.002	0.000
Non‐urgent A&E attendances								
Level^a^	−0.889*	0.433	−0.267	0.820	−0.472*	—	—	—
Age‐related inequality	0.000	−0.001*	0.000	0.000	0.000	—	—	0.000
Socioeconomic‐related inequality	0.000	0.000	0.000	0.000	0.000	0.000	0.000	0.000
Potentially preventable hospital admissions								
Level^a^	−0.051	−0.022	0.005	0.016	0.059	—	—	—

*Note:* Panel (A) refers to the main analysis of 289 practices, 7 “never‐treated” and 282 that installed the online platform after January 2019 up to June 2021 while studying the implementation of the online access route, assuming unconditional parallel trends, measuring the socioeconomic status based on income‐related deprivation levels and using the generalized concentration index for inequality measurement; Panel (B) refers to the analysis that uses the subsample of 26 practices, 7 “never‐treated” and 19 that installed the online platform after January 2019 up to November 2019; Panel (C) refers to the analysis that uses the subsample of 261 practices, 7 “never‐treated” and 254 that installed the online platform in/after March 2020 up to May 2021; Panel (D) refers to the analysis that assumes conditional parallel trends by adjusting for the number of positive tests for SARS‐CoV‐2 per 1000 registered patients, percentage of registered patients who have 1 or more long‐term conditions, number of registered patients per full‐time equivalent GP and administrative/non‐clinical, number of electronic repeat prescriptions ordered by patients per 1000 registered patients and percentage of qualified and permanent GPs who are 55 years or more; Panel (E) refers to the analysis that examines the adoption (rather than implementation) of the online access route across 286 practices, 149 “never‐treated” and 137 that adopted the online access route in/after March 2020. In this case, we excluded 3 practices that adopted the platform prior to the start of the pandemic, that is, March 2020 to enhance comparability among practices; Panel (F) refers to the analysis that measures socioeconomic status based on employment‐related deprivation levels; Panel (G) refers to the analysis that measures socioeconomic status based on education‐related deprivation levels; Panel (H) refers to the analysis that uses the Erreygers index for inequality measurement. — Not applicable.

^a^ Per 1000 registered patients.

^b^ Other staff providing direct patient care such as pharmacists and allied health professionals.

^*^ *p*‐value < 0.05.

#### 5.2.1 Effect on Healthcare Efficiency

Panel A of Table 2 shows that the online access route has led to 107 additional interactions per 1000 registered patients per month in primary care, with 105 additional asynchronous interactions per 1000 registered patients per month and large uncertainty with respect to synchronous interactions. We find no evidence of an effect on the number of synchronous interactions with the GP and nurse, but there have been 15 additional synchronous interactions per 1000 registered patients per month with other clinical staff, including pharmacists and allied health professionals. We also observe no evidence of an effect across communication modes, with the exception of a small increase in video interactions. Regarding unplanned hospital care activity, the online access route has led to under one less non‐urgent attendance to A&E per 1000 registered patients per month, but there is no evidence of an impact on potentially preventable hospital admissions.

#### 5.2.2 Effect on Healthcare Equity

Panel A of Table 2 shows no evidence of an effect of the online access route on age and socieconomic‐related inequality in synchronous interactions, overall and across healthcare professionals or communication modes (specifically, in person or by telephone). The exception is the significant increase in age‐related inequality in synchronous interactions with other clinical staff but the GP and nurse, suggesting care provided by other clinical staff has been further concentrated among the oldest patients. Lastly, patients from different age groups and income‐deprived areas show the same utilization pattern of A&E due to a non‐urgent condition across treated and control groups.

#### 5.2.3 Heterogeneous Effects Over Time

In Figure 7, Panel (i) shows that the effect of online access route on the overall number of interactions in primary care has increased over time, closely resembling the trend in asynchronous communication. There was a reduction in the effect across all categories after 20 months since the implementation of the online access route, but this effect should not be overinterpreted because it was driven by a few early implementers of the online access route prior to the start of the pandemic. In fact, when restricting the sample to 2020/21, this effect is no longer observed, as per Supporting Information S1: Figure S6. In terms of unplanned hospital care activity, there has been a steady reduction in non‐urgent A&E attendances over time after the implementation of the online access route. Finally, we find that the effect on (ii) age and (iii) socioeconomic‐related inequality in synchronous interactions in primary care and non‐urgent A&E attendances have fluctuated around zero. The exception is the noticeable rise over time in age‐inequality in interactions with other clinical staff, which suggests those interactions have increasingly been concentrated among the oldest patients.

#### 5.2.4 Heterogeneous Effects by Practice's Characteristics

In general, there is no evidence of heterogeneous treatment effects according to practice's characteristics.^6^ Few exceptions include evidence of an increase in synchronous interactions and corresponding age‐related inequality (i.e., concentration of healthcare utilization among the oldest patients) for practices with a higher percentage of patients reporting receptionists to be helpful and reduction in income deprivation‐related inequality (i.e., concentration of healthcare utilization among patients from the lowest socioeconomic group) for practices with a higher number of registered patients. See Supporting Information S1: Tables S3–7 for results.

#### 5.2.5 Robustness Checks

Table 2 also includes the estimates from the following robustness checks.

##### 5.2.5.1 Alternative Time Periods: (B) 2018–19 and (C) 2020–21

In line with findings from the main analysis, both analyses show evidence of an increase in the total number of all but particularly asynchronous interactions. Even prior to 2020, in a world where technology adoption was slow, practices that implemented an online system for patients to get in contact with them in a written format also increased their use of technology to communicate asynchronously with patients via text messaging. As a result, practices with an online access route reported more patient‐provider interactions than practices that only offered traditional access routes even before 2020. We also observe an increase in the age‐related inequality in synchronous interactions with other clinical staff (i.e., greater concentration among the oldest patients). The increase in the total number of synchronous interactions with other clinical staff and video interactions is only observed in (C) 2020‐21. Nevertheless, there is no longer evidence of a reduction in the total number of non‐urgent A&E attendances. New findings include an increase of 0.009 in age‐related inequality in telephone interactions (i.e., greater concentration among the oldest patients) in (C) 2020‐21, and an increase of 22 face‐to‐face interactions with the GP along with a reduction of 0.001 in age‐related inequality in non‐urgent A&E attendances (i.e., greater concentration among the youngest patients) in (B) 2018‐19.

##### 5.2.5.2 (D) Conditional Parallel Trends

In this analysis, we also find an increase in the total number of all but particularly asynchronous interactions. In this case, we observe an increase of 17 telephone interactions after the implementation of the online access route. However, there is no longer evidence of an increase in the total number and age‐related inequality in synchronous interactions with other clinical staff or total number of video interactions, and a reduction in the total number of non‐urgent A&E attendances.

##### 5.2.5.3 Different Version of Treatment: (E) Adoption

In accordance with previous findings, results from this analysis also show evidence of an increase in the total number of all but particularly asynchronous interactions and a reduction in the total number of non‐urgent A&E attendances. In contrast to the main analysis, there is no longer evidence of an increase in the total number and age‐related inequality in synchronous interactions with other clinical staff. Instead, there is evidence of a reduction of one video interaction per 1000 registered patients per month.

##### 5.2.5.4 Patient's Socioeconomic Status Based on (F) Employment and (G) Education‐Related Deprivation Levels

The results are generally similar when using income, employment or education‐related deprivation levels to measure the patient's socioeconomic status, with three exceptions. When patient's socioeconomic status is measured based on employment‐related deprivation levels, there is evidence of an increase of 0.003 in socioeconomic‐related inequality in synchronous interactions both across healthcare professionals and with the GP in particular. When patient's socioeconomic status is measured based on education‐related deprivation levels, there is evidence of an increase of 0.002 in socioeconomic‐related inequality in synchronous interactions with the nurse. These results suggest that the implementation of the online access route has led to a lesser concentration of healthcare utilization among patients from the lowest socioeconomic group. It is worth noting, however, that despite “statistically significant” (*p*‐value < 0.05), the magnitude of all estimated effects is relatively small.

##### 5.2.5.5 Alternative Measure of Inequality: (H) Erreygers Index

The results from this analysis are in accordance with those based on the generalized concentration index.

## 6 Discussion

The digitalization of access to primary care in the English NHS provides an important case study for examining the potential of technology, implemented at scale to tackle the problem of waiting times in this setting. The central question is whether a potential efficiency improvement might be achieved at the expense of lower provision of primary care services to patients from older or lower socioeconomic groups. This study provides robust evidence on this potential equity‐efficiency trade‐off by exploiting the differential timing of online platforms' implementation between 2019 and 2020 across practices in NW London. We find that the implementation of the online access route to primary care supports efficiency improvement by increasing all but particularly asynchronous interactions in primary care along with a small reduction (at worst, no changes) in non‐urgent visits to A&E for a similar average change in primary care resources across treated and control practices. In addition, the online access route shows no impact on healthcare equity since there is no evidence of an increase in age and socioeconomic‐related inequality in synchronous interactions in primary care or non‐urgent A&E attendances for a similar average change in clinical need across treated and control practices.

It is important to acknowledge the potential confounding effect of COVID‐19 on studies like the present one that include data after March 2020. In particular, the fear of contracting COVID‐19 is likely to have led patients to avoid hospital services. In addition, younger groups who typically need primary care services sporadically were more likely to require the attention of primary care professionals if infected by the virus. Thus, COVID‐19 pandemic could have reduced unplanned hospital care activity and decreased age‐related inequality (pro‐young) in primary care interactions directly. However, the fact that COVID‐19 impacted both treated and control practices in a similar fashion (see Section 4.1.1 for a detailed examination of parallel trends assumption) and that DiD identification strategy can handle time‐invariant confounding give us confidence about the validity of our findings. Moreover, results are generally observed across several robustness checks, including the analysis that assessed the effect of the online access route to primary care before and after the pandemic separately. More importantly, the few differences observed—such as a reduction in the magnitude (albeit still significant) of patient‐provider interactions in primary care and no evidence of a change in non‐urgent A&E attendances in some cases—do not change the main message from this study. The evidence still points toward an increase in primary care provision after the introduction of the online channel. Notably, this increase comes at no detriment to patients from older and lower socioeconomic groups.

Even though we were not able to estimate inequality in asynchronous interactions, it is likely that many of these interactions are provided to younger patients with appropriate digital skills and resources (Jung and Padman 2014; Weiss et al. 2018; Rodgers et al. 2019; Zanaboni and Fagerlund 2020; Honeyman et al. 2020; Ortega et al. 2020; Darley et al. 2022). However, the key concern in the community is not about the likely increase in asynchronous interactions among patients from younger and high socioeconomic groups. Instead, the key concern is that the elderly and patients from lower socioeconomic groups who tend to use more traditional access routes to primary care could have less opportunities to speak or meet in person with a clinician, if patients using the online channel were offered these type of interactions first, irrespective of clinical need. What we find is no evidence supporting changes to age or socioeconomic‐related inequality in synchronous interactions due to the intervention.

When focusing on efficiency, we assume that any increase in the volume of primary care activity using the same resources represents an improvement in efficiency. However, this would not be the case if this increase was driven by low‐value activity. While most of the increase in patient‐provider interactions stems from asynchronous interactions, these interactions can offer both low or high value to patients depending on the context. For example, asynchronous interactions can be particularly helpful for certain patient groups such as those with physical disabilities (Bakhai and Atherton 2021) and to manage certain health problems such as chronic conditions (Khoong 2022) or discuss more sensitive health‐related topics (Bakhai and Atherton 2021). Yet, if multiple asynchronous interactions were provided for the same patient request and ultimately a synchronous consultation was offered, this care could be perceived as low value. In this work, however, it is not possible to distinguish between high‐ and low‐value patient‐provider interactions in primary care. Nevertheless, given the reduction in satisfaction with access to primary care reported in the 2022 GP Patient Survey (Ipsos 2022), any increase in the volume of primary care activity is treated as an improvement.

It is also worth highlighting that if there were differences over time between treated and control practices with respect to workforce capacity, then the increase in patient‐provider interactions might not reflect a more efficient utilization of existing resources but the increase in workforce capacity. Even though data support a similar number of GPs and administrative staff across treated and control practices over time, limited data reported for other primary care professionals at the time of the study did not allow us to run similar checks. Nevertheless, similar payments received per patient across treated and control practices over time support the assumption of similar number of other primary care professionals across treated and control practices over time. Furthermore, by drawing on the work by General Medical Council (2021), it also seems that the general practice workforce worked extra hours to cope with the rise in workload. While this finding supports the idea of doing more with the same resources, the increase in working hours for staff also has implications for patient safety and job burnout and should be the focus of further research.

While it might be possible to generalize the current findings to other major cities in England beyond London, it is worth noting that results might differ in rural areas. Future research should examine the generalizability of current findings to the whole country and perhaps other countries, in particular, those facing the same problem of excessive waiting times in primary care. Moreover, the study timeframe includes a period of drastic changes in the NHS as part of the response to the COVID‐19 pandemic. While many changes became part of routine care, other processes were only temporary. For example, the online system was initially available 24/7, but afterward some practices started to restrict access to working hours during weekdays only. Moreover, during the pandemic most practices limited in‐person requests, with some practices promoting the telephone while others, the online system as their recommended access channel. As a learning health system, the NHS should continue to learn about the impact of having an online access route to primary care and adjust the operational workflow accordingly. It will also be important to extend this evaluation to other outcomes such as burnout among primary healthcare professionals and continuity of care for patients. In summary, despite the need to continuously monitor potential safety incidents and adverse events, initial evidence provided in this study shows that there is potential for the digitalization of access to primary care to improve the utilization of healthcare services for the patient population.

## Ethics Statement

This work was approved by the NW London Sub‐Data Research Access Group (SDRAG Ref ID‐206). All data used in this work were fully anonymized before analysis.

## Conflicts of Interest

The authors declare no conflicts of interest.

## Supporting information

Supporting Information S1

## Data Availability

To access the Whole Systems Integrated Care (WSIC) de‐identified dataset refer to https://www.nwlondonicb.nhs.uk/professionals/whole‐systems‐integrated‐care‐wsic/information‐de‐identified‐users. This work also contains public sector information from NHS Digital, Ipsos GP Patient Survey, Department for Leveling Up, Housing and Communities, Office of National Statistics, Care Quality Commission, and Ofcom under the Open Government License v3.0.

## Endnotes

^1^
January 2019 is the last month before the first practices implement the online platform.

^2^
The process of defining and measuring healthcare utilization in primary care was developed with input from the WSIC and NHS Digital's primary care teams while following other official guidance (NHS England and NHS Improvement and the British Medical Association 2020; NHS England 2021; NHS Digital 2023b).

^3^
Video includes audio only through a digital medium.

^4^
These forbidden comparisons occur when standard “two‐way fixed effects” (TWFE) regressions are used for estimation in settings where different groups are exposed to the treatment at different points in time (current case) and are particularly problematic when there is treatment effect heterogeneity over time because the difference in pre and post‐treatment outcomes for the already‐treated (control) group will include the difference in the average treatment effect over time as well (Borusyak and Jaravel 2016; De Chaisemartin and D’Haultfœuille 2020; Sun and Abraham 2021; Goodman‐Bacon 2021). A. C. Baker et al. (2022) demonstrated the practical implications of this finding by replicating the analysis of previously published studies using estimators that allow for treatment effect heterogeneity over time—including Callaway and Sant’Anna (2021)—and standard TWFE regressions and finding “substantially” different results. In the context of the main analysis of our study, the Bacon decomposition (Goodman‐Bacon 2021) shows that around 31% of all estimates underlying the overall ATT estimate via a standard TWFE regression would be derived from forbidden comparisons. When assessing the impact of the intervention on the level of all patient‐provider interactions, forbidden comparisons would lead to an underestimation of the ATT. For this reason, we opted for the Callaway and Sant’Anna (2021) approach which avoids any biases arising from using such comparisons.

^5^
When assuming conditional parallel trends, we also assume overlap which means that practices with similar characteristics have a positive probability of implementing the online access route.

^6^
As part of the robustness checks, we also find no evidence of a change in synchronous interactions due to the intervention in practices serving an older/younger patient population and/or from a lower/higher socioeconomic group. See Supplementary Table S8 for results.
