# Supplementary material for: Digitalization of Access to Primary Care: Is There an Equity‐Efficiency Trade‐Off?
Source: Health Econ. 2025 Jul 17;34(10):1943–62. doi: 10.1002/hec.70014 (PMC12412092; doi:10.1002/hec.70014)
Supplement: Supplementary file 1 — Supporting Information S1 [file HEC-34-1943-s001.pdf]

Supplementary material

Digitalisation of access to primary care: is there an equity-efficiency trade-off?

April 30, 2025

Supplementary Table S1: Publicly available data used in this study.

Category	Source
Other patient-initiated online care services	General Practice data hub†
Deprivation-related information	Department for Levelling Up, Housing and Communities‡
Connectivity-related information	Ofcom§
Practices' business structure/ inspection ratings	Care Quality Commission¶
Finance-related information	NHS Payments to General Practice#
Workforce-related information	General Practice Workforce††
Workforce-related information	GP and GP practice related data‡‡
Patients' experience of their practice	GP Patient Survey§§

Note:

† https://digital.nhs.uk/data-and-information/data-tools-and-services/data-services/general-practice-data-hub

‡ https://opendatacommunities.org/def/concept/folders/themes/societal-wellbeing

§ https://www.ofcom.org.uk/research-and-data/multi-sector-research/infrastructure-research

¶ https://www.cqc.org.uk

# https://digital.nhs.uk/data-and-information/publications/statistical/nhs-payments-to-general-practice

†† https://digital.nhs.uk/data-and-information/publications/statistical/general-and-personal-medical-services

‡‡ https://digital.nhs.uk/services/organisation-data-service/file-downloads/gp-and-gp-practice-related-data

§§ https://gp-patient.co.uk

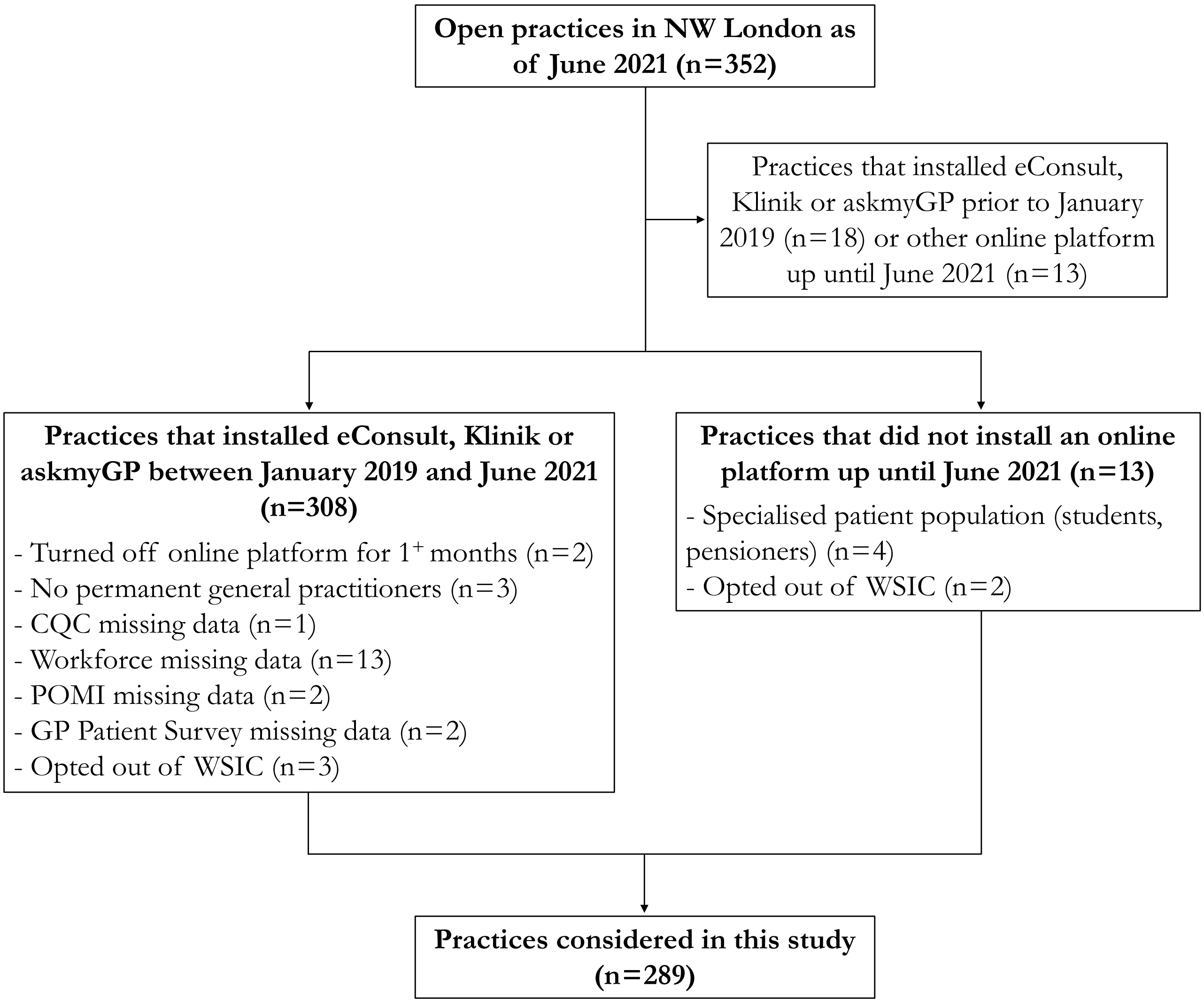

Supplementary Figure S1: Eligibility criteria. *Note:* CQC indicates Care Quality Commission; NW, North West; n, number; POMI, Patient Online Management Information; WSIC, Whole Systems Integrated Care.

Supplementary Table S2: Characteristics of the study sample of 289 practices in NW London as of January 2019

Variable	N (%)	Min	Max	Median	Mean
**Patients' demographics**					
% of reg. patients who are female	289 (100)	32.53	61.85	49.15	48.53
% of reg. patients who are 65+ years	289 (100)	0.08	23.96	11.56	11.73
Average income decile of reg. patients	289 (100)	1.90	9.01	4.70	4.87
Average employment decile of reg. patients	289 (100)	2.27	9.11	5.77	5.79
Average education decile of reg. patients	289 (100)	3.40	9.89	7.07	6.95
Average IDAOPI decile of reg. patients	289 (100)	1.36	8.60	3.48	3.80
**Patients' health status**					
% of reg. patients who have 1+ chronic conditions	289 (100)	6.34	40.45	27.82	27.48
Number of positive tests for SARS-CoV-2 per 1000 reg. patients	0.00	0.00	0.00	0.00	
**Internet connectivity at patients' residential area**					
Average download speed (Mbit/s) at the residential area of reg. patients	289 (100)	29.48	87.60	70.13	65.86
**Patients' digital literacy**					
Number of electronic repeat prescriptions ordered by patients per 1000 reg. patients	289 (100)	0.00	138.61	31.35	34.56
**Practice's organisational characteristics**					
Average number of reg. patients[a]	289 (100)	1584	22258	6285	6937
Business structure (0-Individual; 1-Partnership/Organisation)	[66 (23); 223 (77)]	-	-	-	-
Average payment (£) received per weighted (*i.e.*, standardised) patient	289 (100)	78.92	367.80	147.89	152.03
**Workforce**					
Number of reg. patients per full-time equivalent GP[a]	289 (100)	346	15358	2135	2342
Number of reg. patients per full-time equivalent administrative/non-clinical[a]	289 (100)	306	11859	1136	1339
% of qualified and permanent GPs who are female	289 (100)	0.00	100.00	50.00	51.56
% of qualified and permanent GPs who are 55+ years	289 (100)	0.00	100.00	28.57	35.08
Training practice (0-No; 1-Yes)	[204 (71); 85 (29)]	-	-	-	-
**Internet connectivity at the practice**					
Average download speed (Mbit/s) at the practice	289 (100)	4.50	129.30	63.00	62.41
**Practice's QOF achievement**					
% of available QOF points that were achieved	289 (100)	63.21	100.00	97.91	96.42
**Practice's CQC rating**					
CQC rating on safety (0-Requires improvement/Inadequate; 1-Good/Outstanding)	[22 (8); 266 (92)]	-	-	-	-
CQC rating on effectiveness (0-Requires improvement/Inadequate; 1-Good/Outstanding)	[16 (6); 272 (94)]	-	-	-	-

Table S2 Characteristics of the study sample of 289 practices in NW London as of January 2019 (Continued)

Variable	N (%)	Min	Max	Median	Mean
CQC rating on responsiveness (0-Requires improvement/Inadequate; 1-Good/Outstanding)	[7 (2); 281 (97)]	-	-	-	-
CQC rating on caring (0-Requires improvement/Inadequate; 1-Good/Outstanding)	[10 (3); 278 (96)]	-	-	-	-
CQC rating on well-led (0-Requires improvement/Inadequate; 1-Good/Outstanding)	[15 (5); 273 (94)]	-	-	-	-
**Patients' experience of the practice**					
% of reg. patients who reported to be easy† to contact the practice by phone	289 (100)	33.37	100.00	77.27	74.76
% of reg. patients who reported receptionists to be helpful†	289 (100)	59.74	100.00	87.88	87.16
% of reg. patients who reported to be easy† to use the practice's website	289 (100)	35.01	96.79	75.33	74.12
% of reg. patients who reported to be satisfied† with practice's appointment times	289 (100)	43.21	92.35	65.46	65.87

Note:

CQC indicates Care Quality Commission; GP, general practitioner; IDAOPI, Income Deprivation Affecting Older (60^{+} years) People Index; NW, North West; QOF, Quality and Outcomes Framework; reg., registered

[a] Round number to next lowest integer

- Not applicable

† includes "fairly" or "very" as per Ipsos General Practice Patient Survey (https://gp-patient.co.uk)

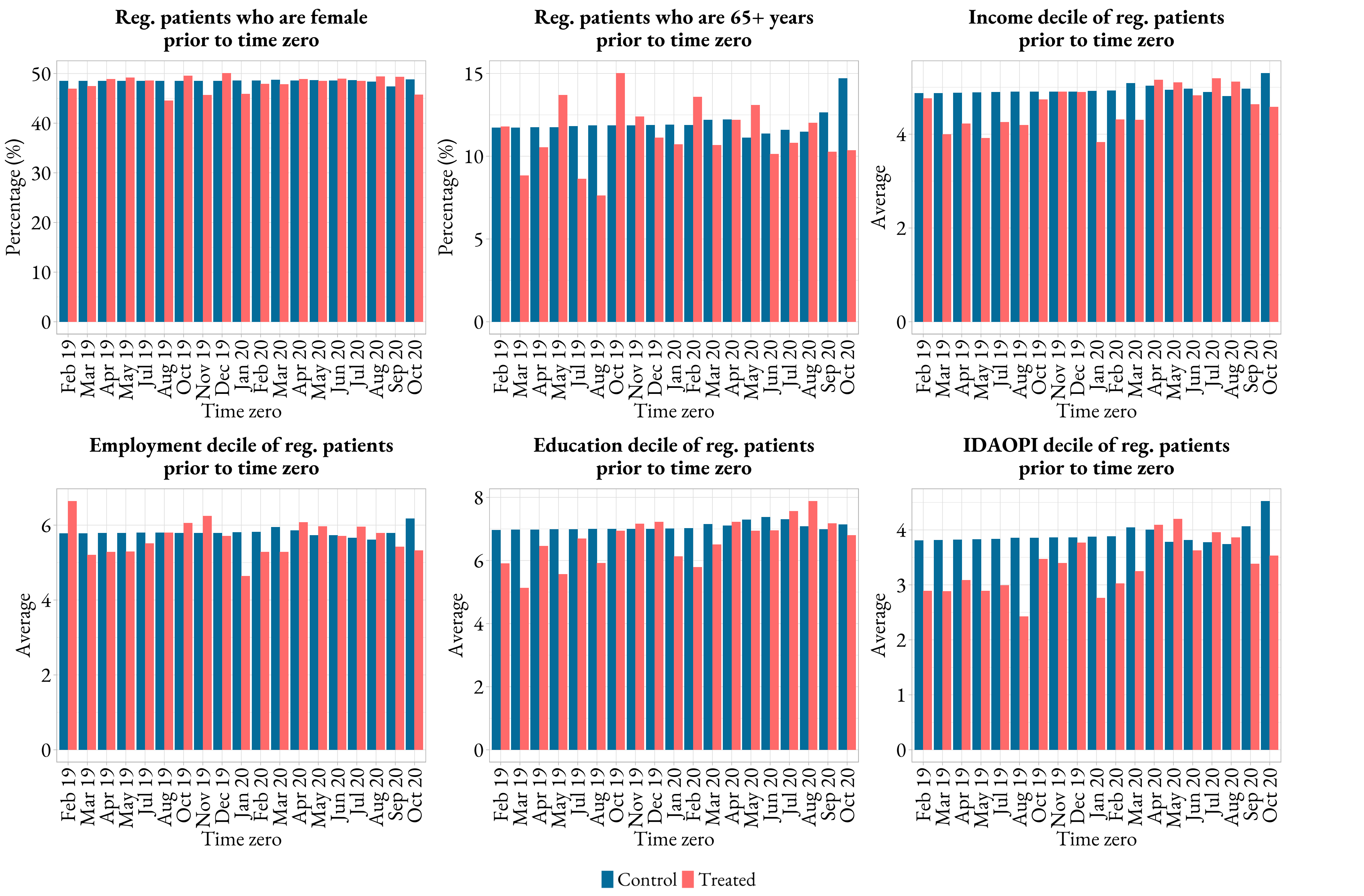

Supplementary Figure S2: Pre-treatment covariates across treated and control groups for when post-treatment time equals time zero period and treatment is the implementation of the online access route. *Note:* IDAOPI indicates Income Deprivation Affecting Older (60^{+} years) People Index; reg., registered.

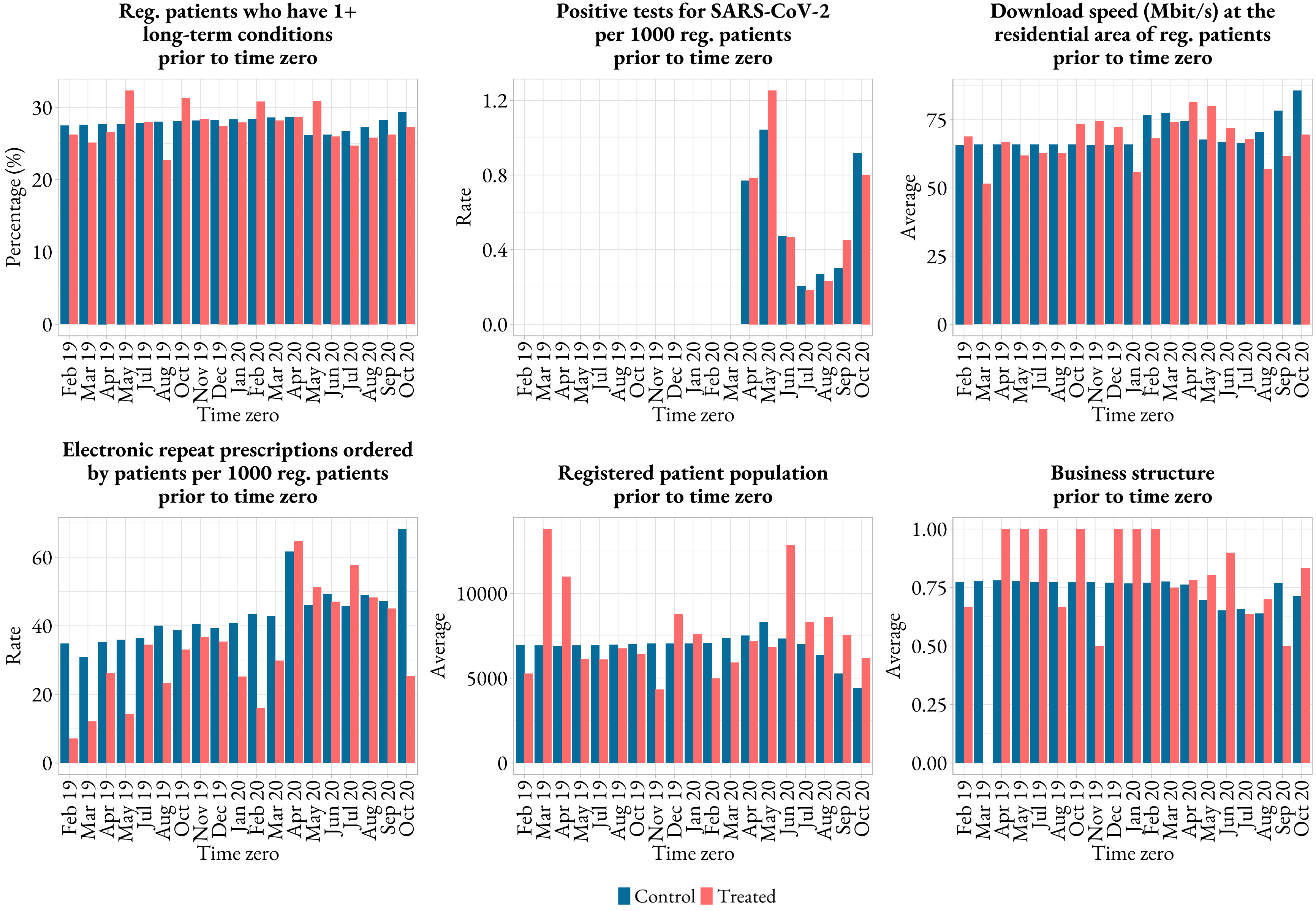

Supplementary Figure S2: Pre-treatment covariates across treated and control groups for when post-treatment time equals time zero period and treatment is the implementation of the online access route. *Note:* Reg. indicates registered. (Continued)

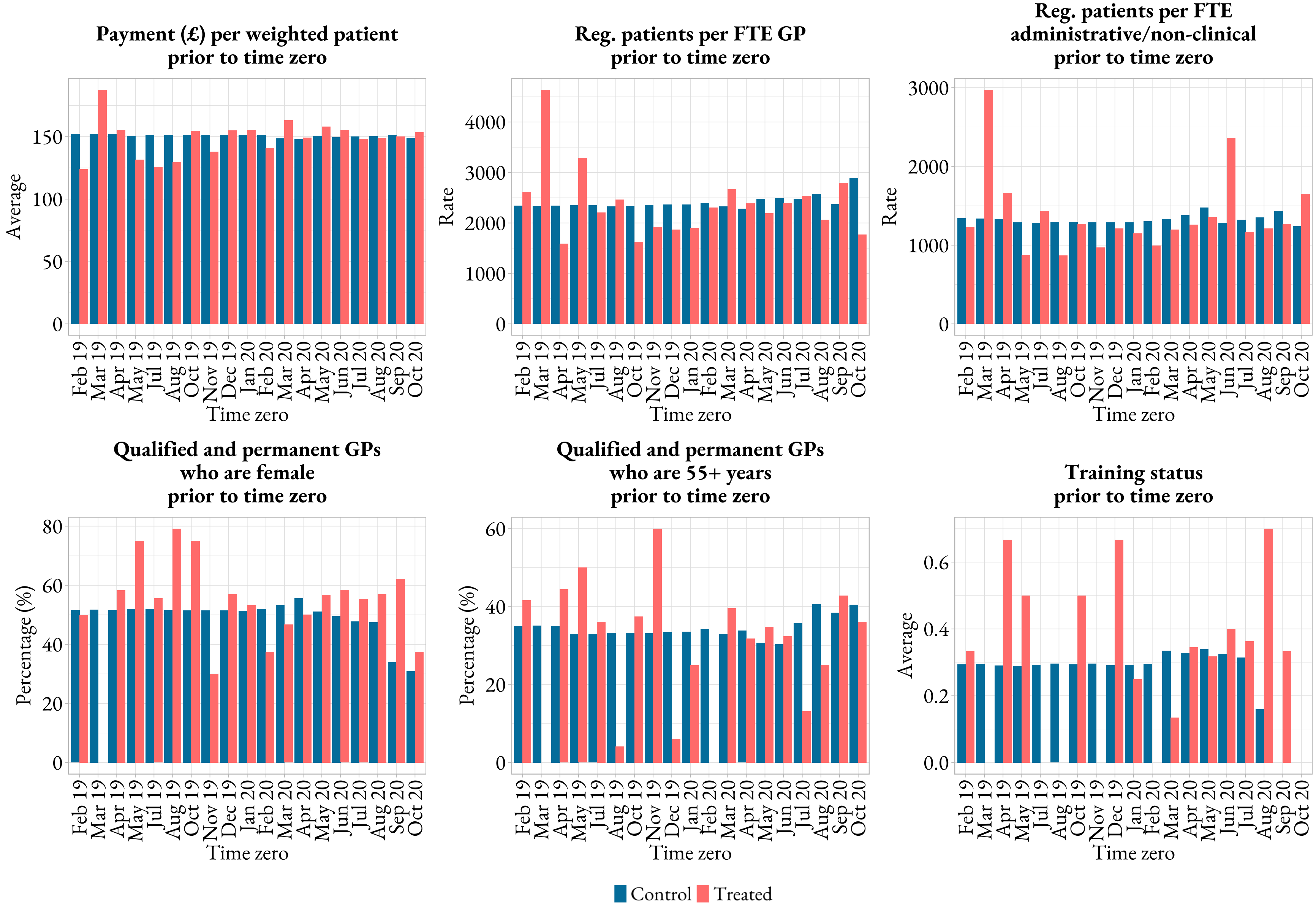

Supplementary Figure S2: Pre-treatment covariates across treated and control groups for when post-treatment time equals time zero period and treatment is the implementation of the online access route. *Note:* FTE indicates full-time equivalent; GP, General Practitioner; reg., registered. (Continued)

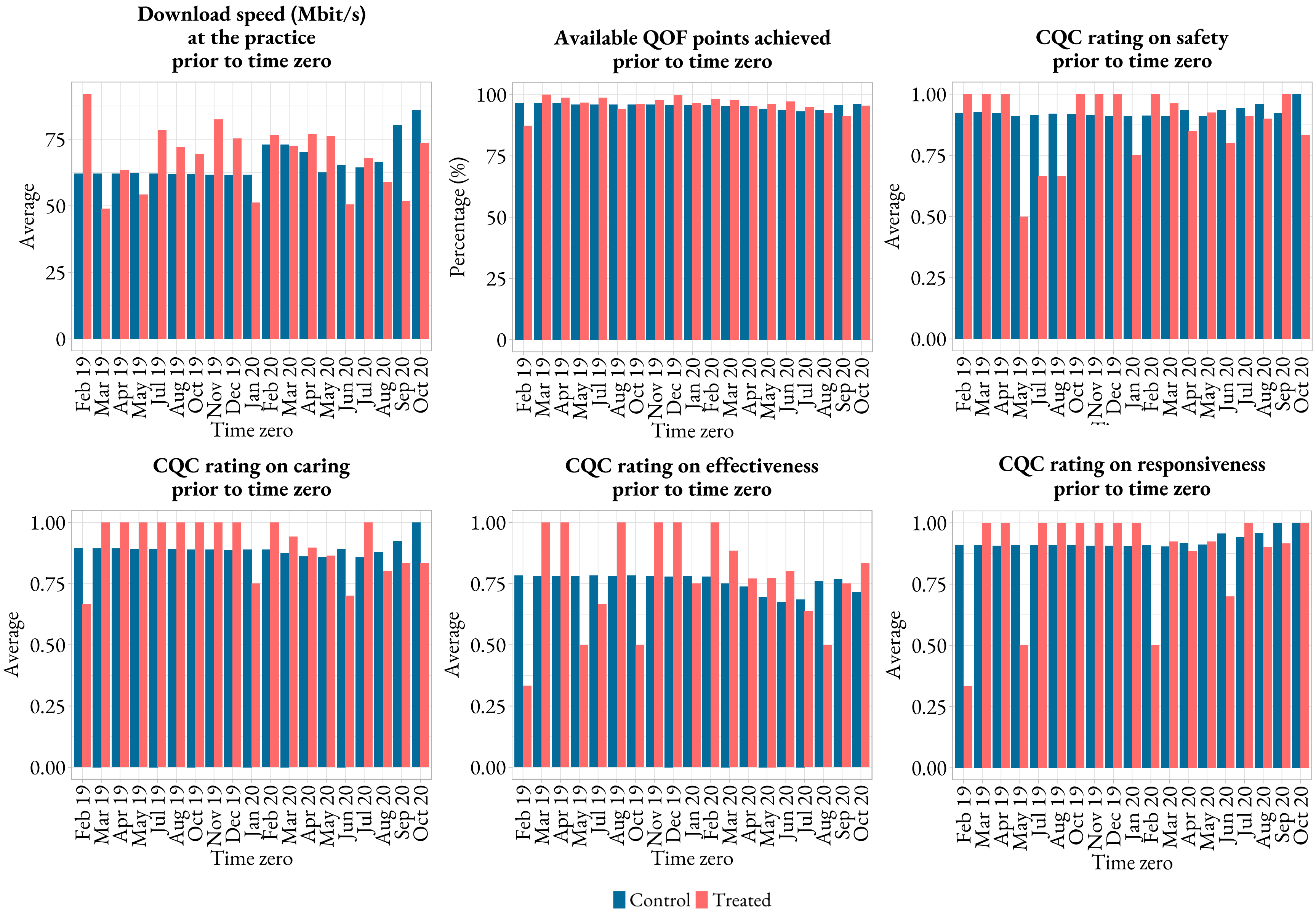

Supplementary Figure S2: Pre-treatment covariates across treated and control groups for when post-treatment time equals time zero period and treatment is the implementation of the online access route. *Note:* QOF indicates Quality and Outcomes Framework; CQC, Care Quality Commission; reg., registered. (Continued)

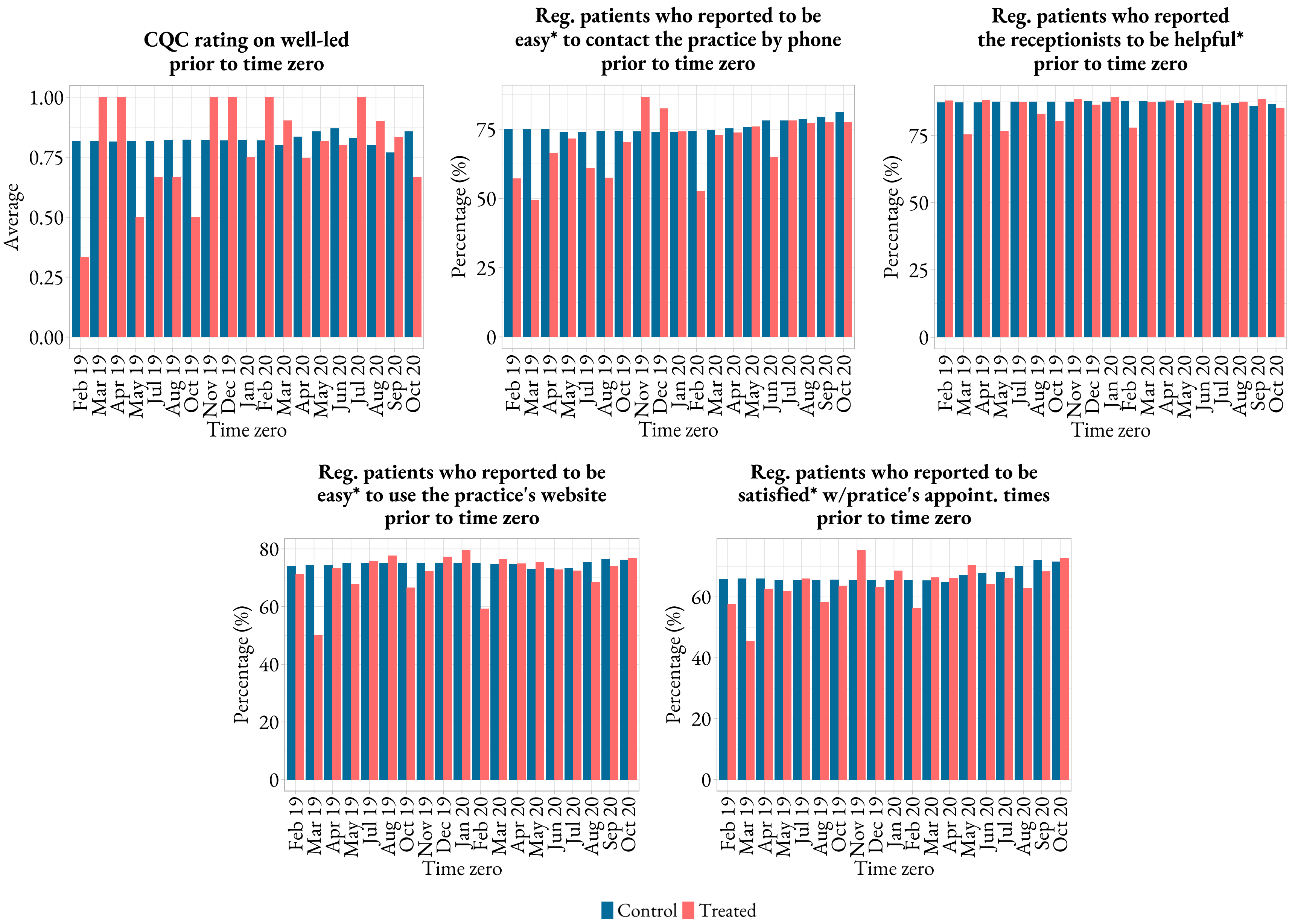

Supplementary Figure S2: Pre-treatment covariates across treated and control groups for when post-treatment time equals time zero period and treatment is the implementation of the online access route. *Note:* appoint. indicates appointment; CQC, Care Quality Commission; reg., registered; w/, with; * includes "fairly" or "very" as per Ipsos General Practice Patient Survey (https://gp-patient.co.uk). (Continued)

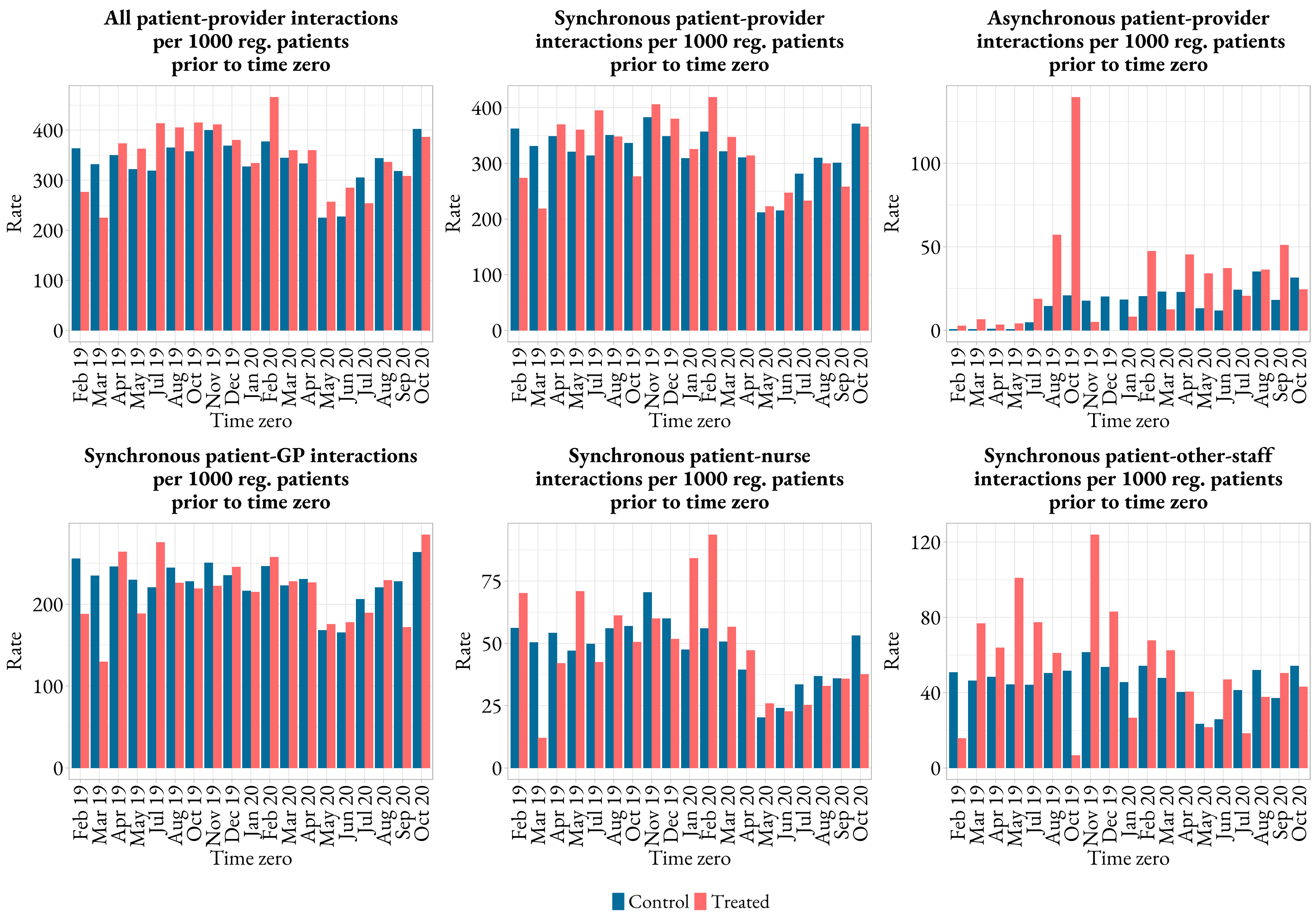

Supplementary Figure S3: Pre-treatment outcomes across treated and control groups for when post-treatment time equals time zero period and treatment is the implementation of the online access route. *Note:* GP indicates General Practitioner.

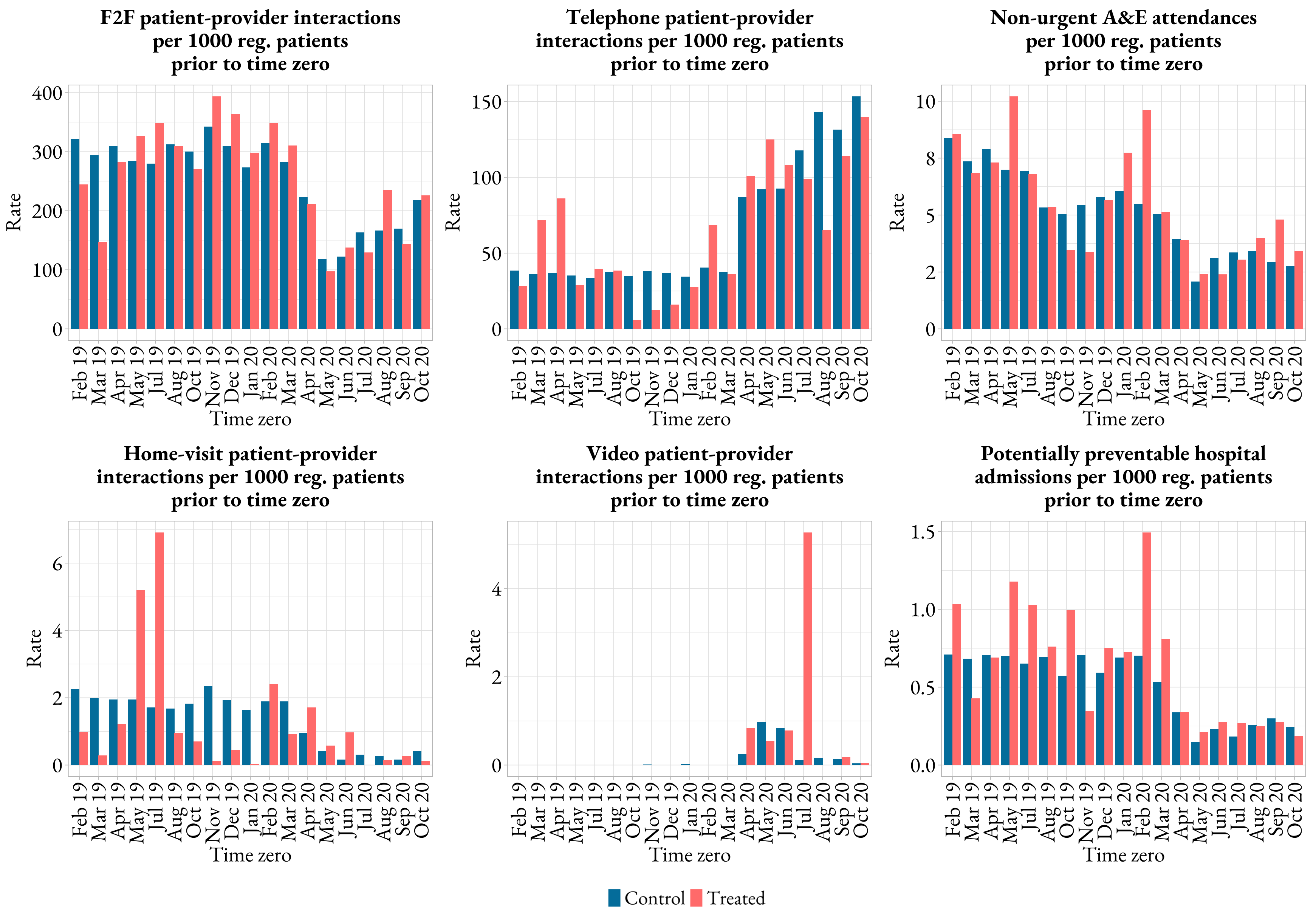

Supplementary Figure S3: Pre-treatment outcomes across treated and control groups for when post-treatment time equals time zero period and treatment is the implementation of the online access route. *Note:* F2F indicates face-to-face; A&E, Accident & Emergency department. (Continued)

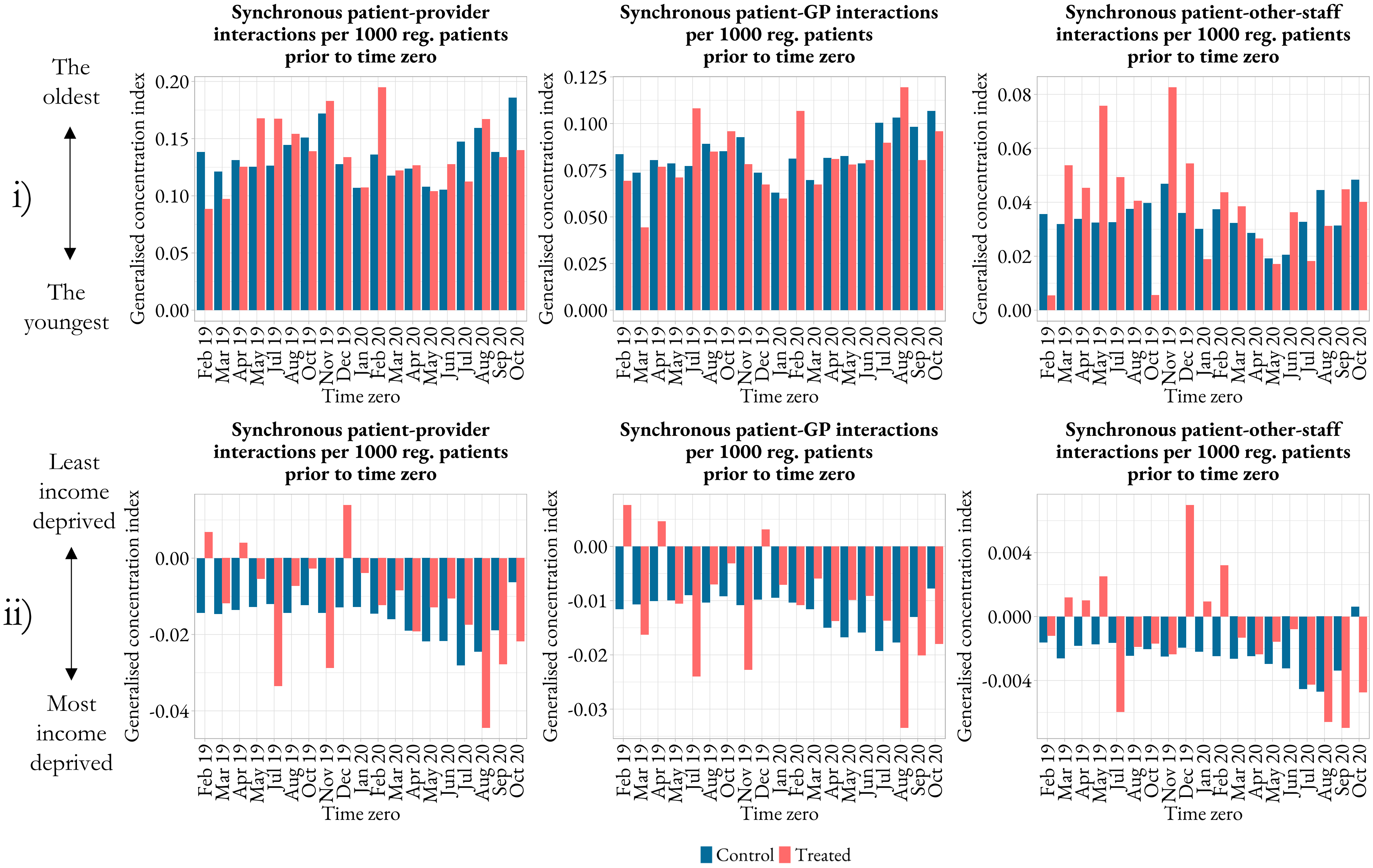

Supplementary Figure S3: Pre-treatment outcomes across treated and control groups for when post-treatment time equals time zero period and treatment is the implementation of the online access route. *Note:* GP indicates General Practitioner. (Continued)

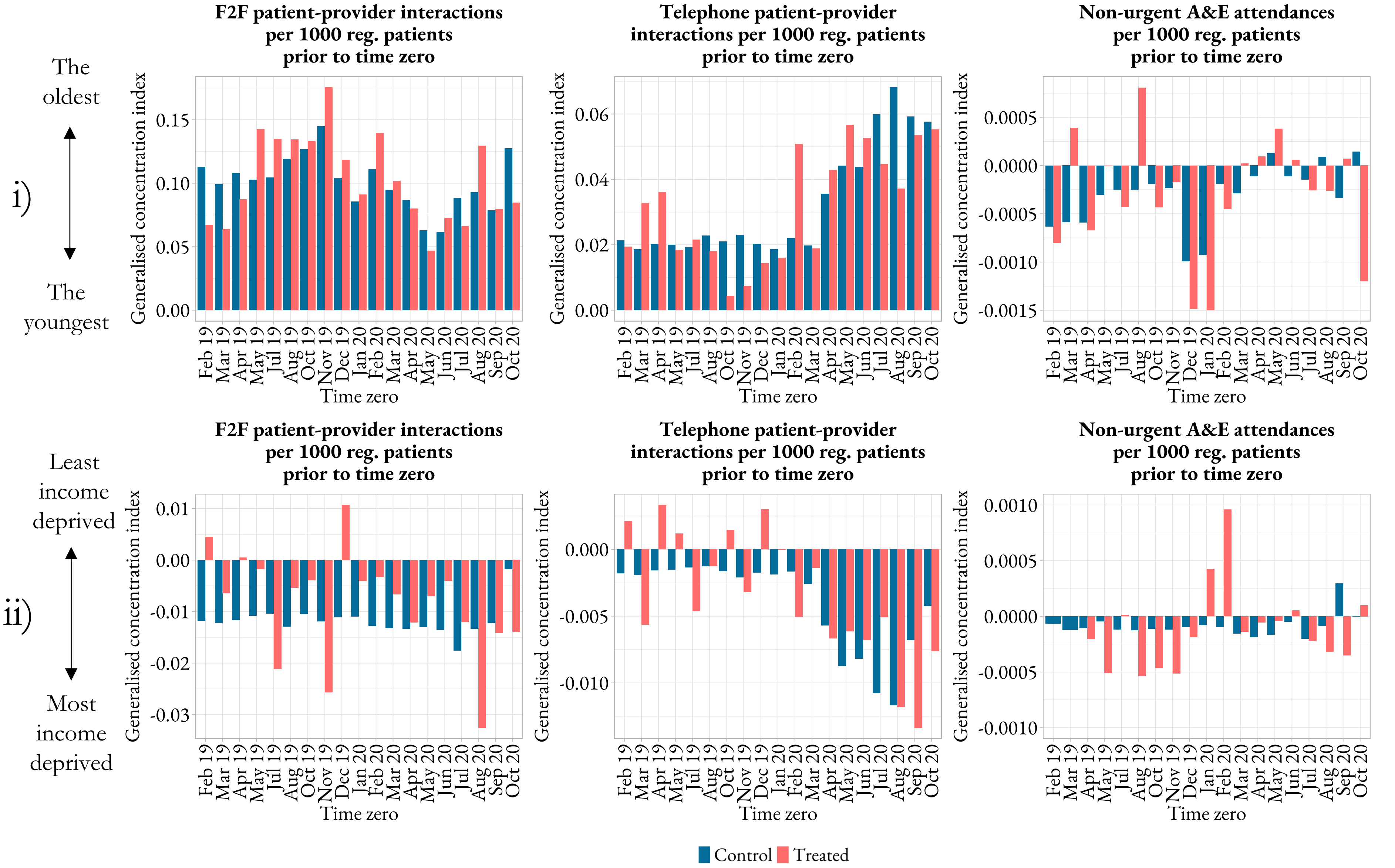

Supplementary Figure S3: Pre-treatment outcomes across treated and control groups for when post-treatment time equals time zero period and treatment is the implementation of the online access route. *Note:* F2F indicates face-to-face; A&E, Accident & Emergency department. (Continued)

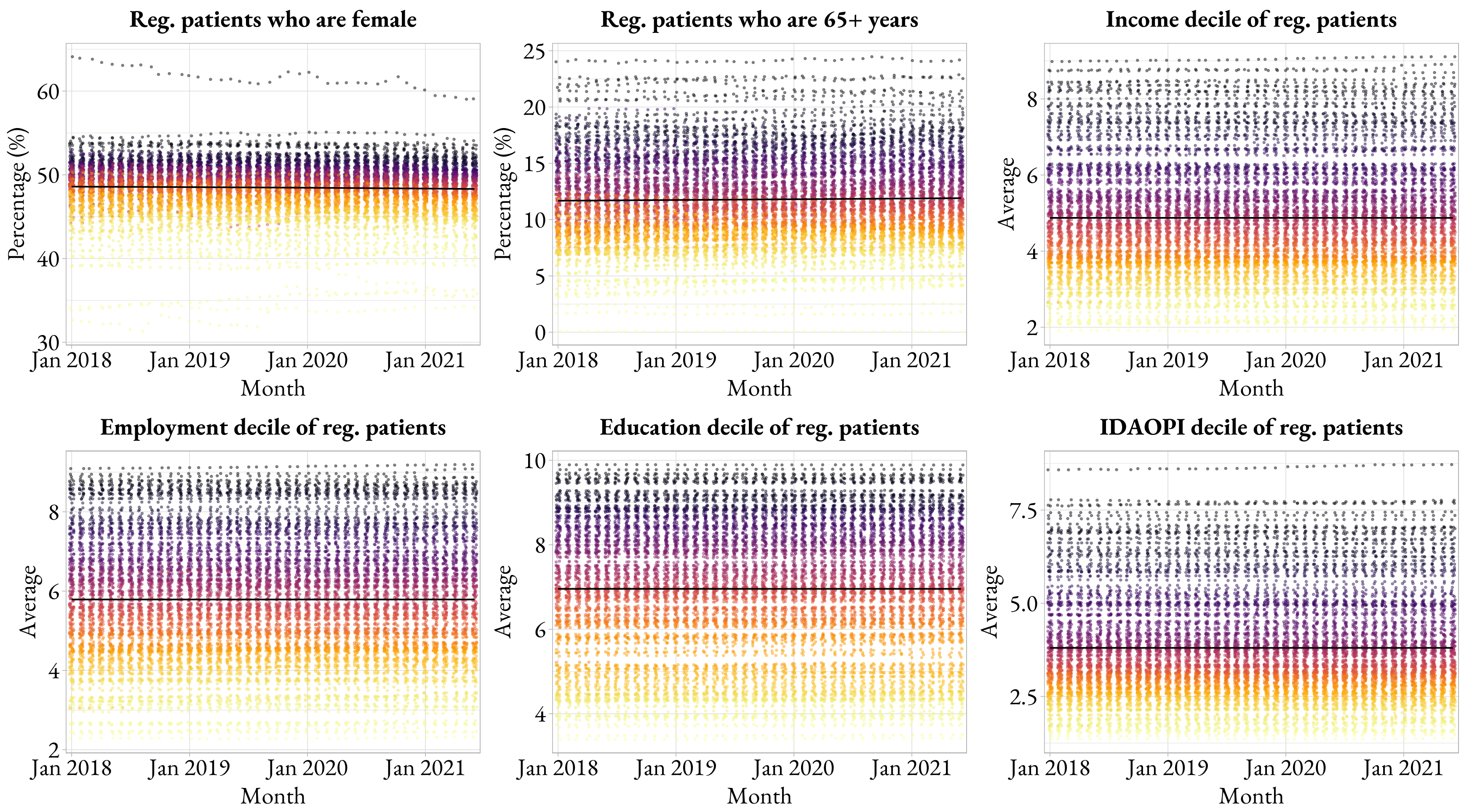

Supplementary Figure S4: Covariates over time for all practices in the sample and their patient population. *Note:* IDAOPI indicates Income Deprivation Affecting Older (60^+ years) People Index; reg., registered. Each dot is a practice; practices are coloured according to their position in each covariate distribution as of June 2021, from yellow (practices with lowest values) to dark grey (practices with highest values).

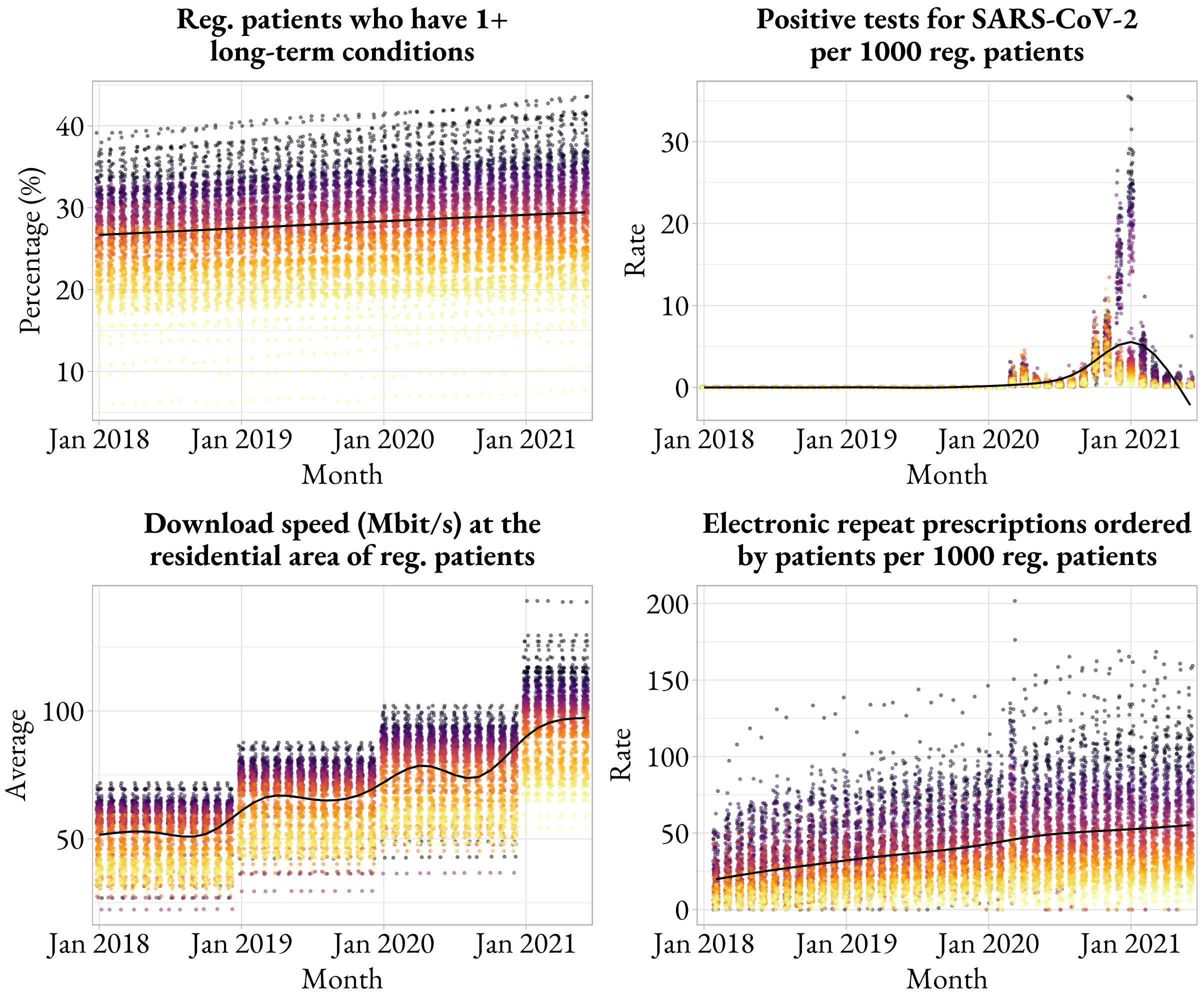

Supplementary Figure S4: Covariates over time for all practices in the sample and their patient population. *Note:* Reg. indicates registered. Each dot is a practice; practices are coloured according to their position in each covariate distribution as of June 2021 (with the exception of positive tests for SARS-CoV-2 per 1000 reg. patients; for this variable, January 2021 was used as reference because that was when maximum rates and overall variation were registered at the time of the study), from yellow (practices with lowest values) to dark grey (practices with highest values). (Continued)

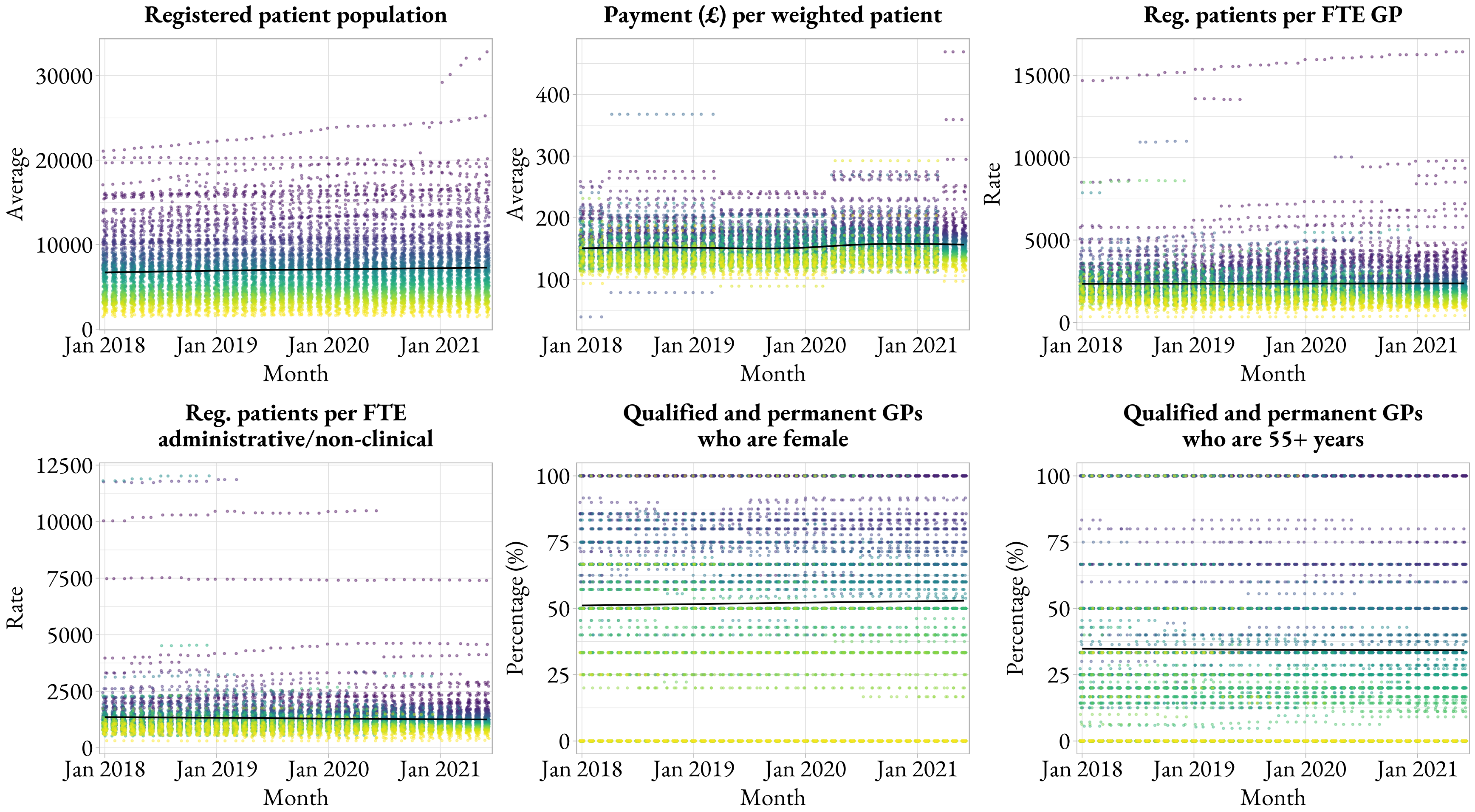

Supplementary Figure S4: Covariates over time for all practices in the sample and their patient population. *Note:* FTE indicates full-time equivalent; GP, general practitioner; reg., registered. Each dot is a practice; practices are coloured according to their position in each covariate distribution as of June 2021, from yellow (practices with lowest values) to dark purple (practices with highest values). (Continued)

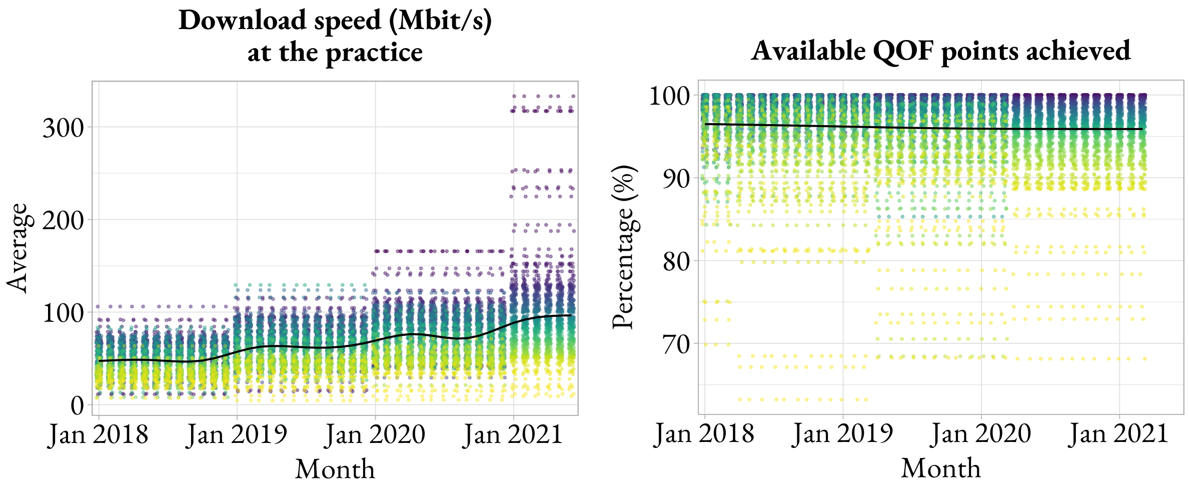

Supplementary Figure S4: Covariates over time for all practices in the sample and their patient population. *Note:* QOF indicates Quality and Outcomes Framework. Each dot is a practice; practices are coloured according to their position in each covariate distribution as of June 2021, from yellow (practices with lowest values) to dark purple (practices with highest values). (Continued)

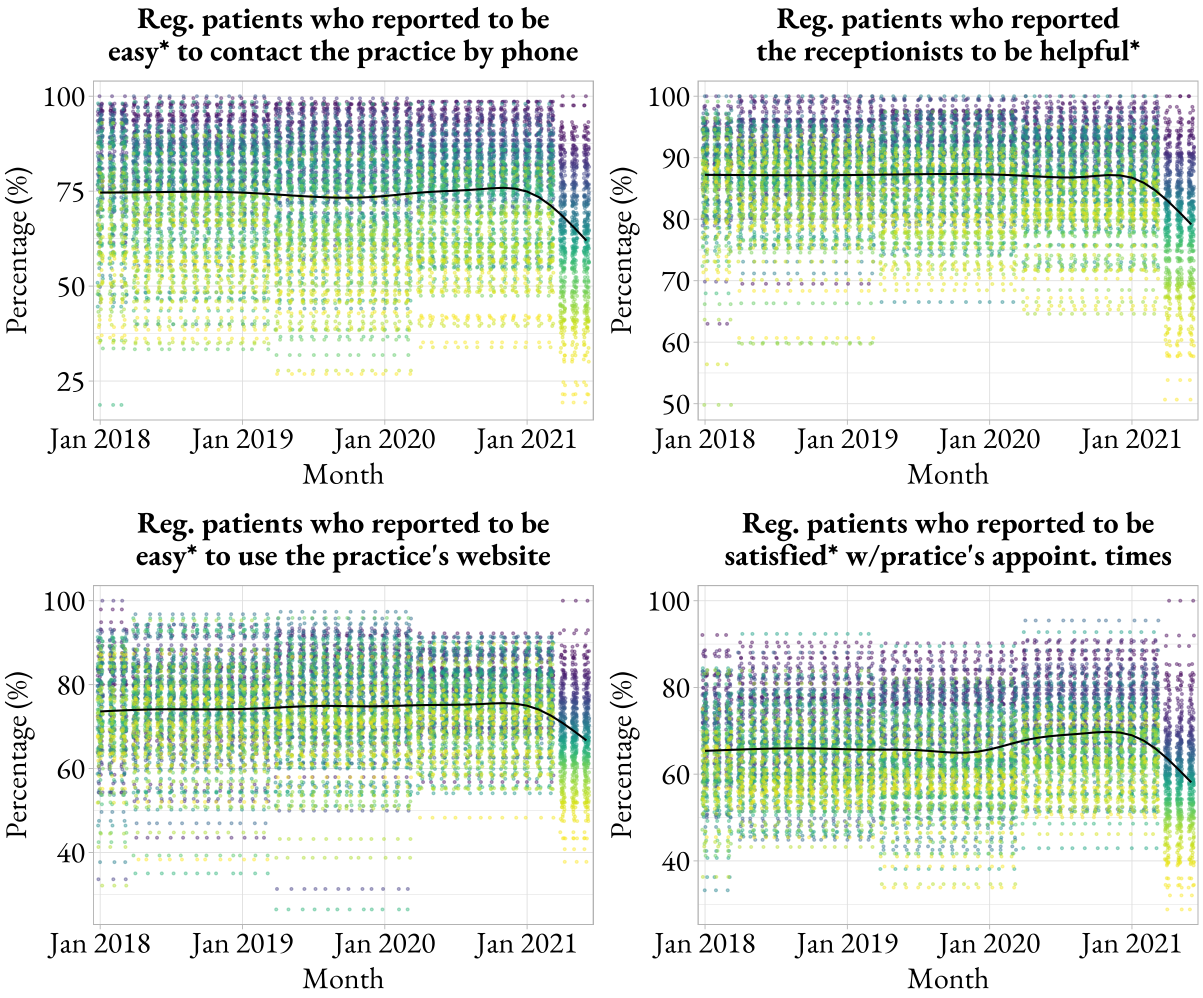

Supplementary Figure S4: Covariates over time for all practices in the sample and their patient population. *Note:* appoint. indicates appointment; reg., registered; w/, with; * includes "fairly" or "very" as per Ipsos General Practice Patient Survey (https://gp-patient.co.uk). Each dot is a practice; practices are coloured according to their position in each covariate distribution as of June 2021, from yellow (practices with lowest values) to dark purple (practices with highest values). (Continued)

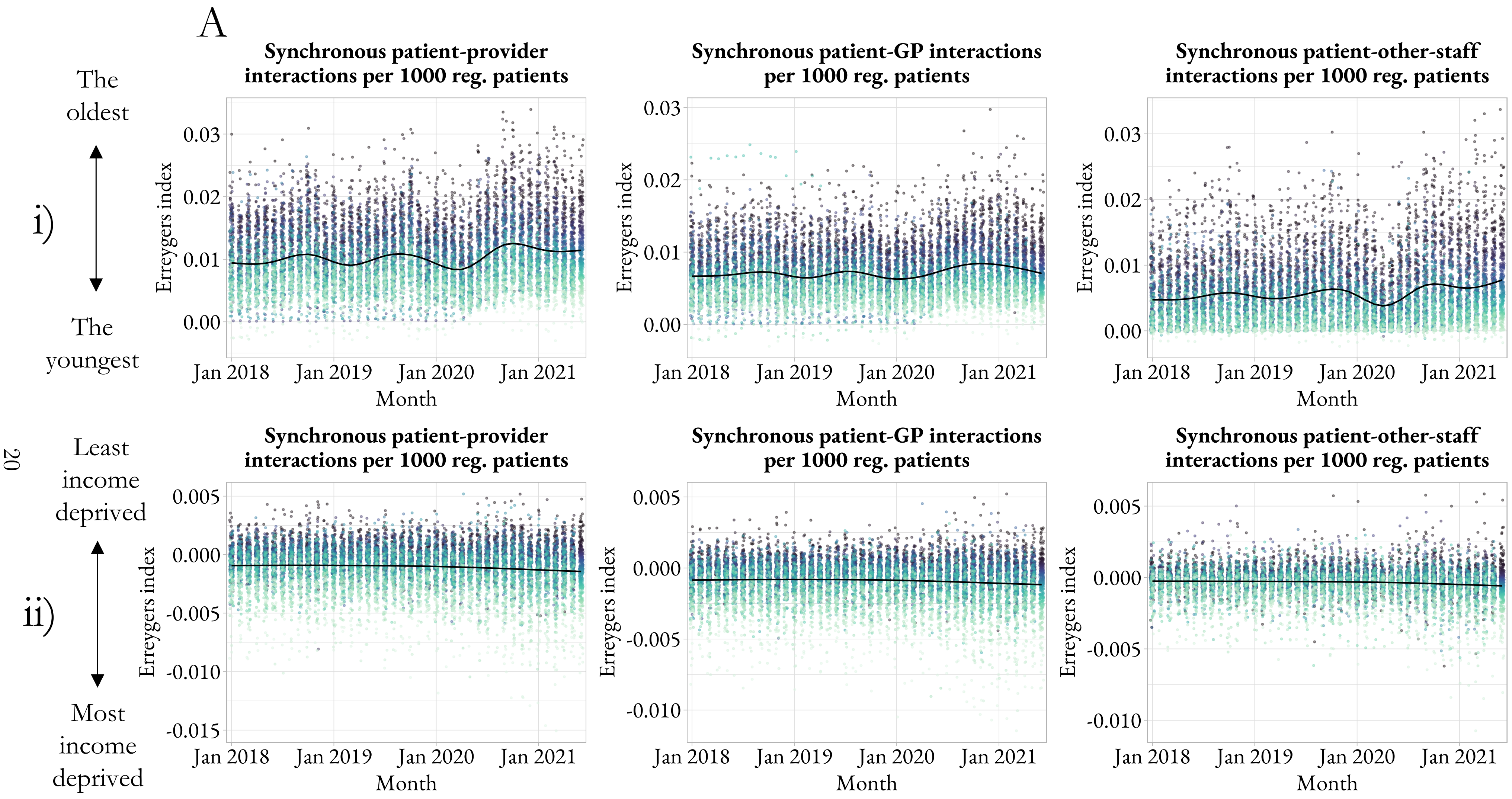

Supplementary Figure S5: Inequality in healthcare utilisation based on the Erreygers index. *Note:* GP indicates General Practitioner; reg., registered. Panels *i)* and *ii)* represent age and socioeconomic-related inequality, respectively. Panel A shows inequality in synchronous interactions in primary care and Panel B depicts inequality in non-urgent non-urgent A&E attendances. The data are between January 2018 and June 2021. Socioeconomic status is based on income-related deprivation levels. Each dot is a practice; practices are coloured according to their position in each outcome distribution as of June 2021, from light green (practices with lowest values) to dark grey (practices with highest values).

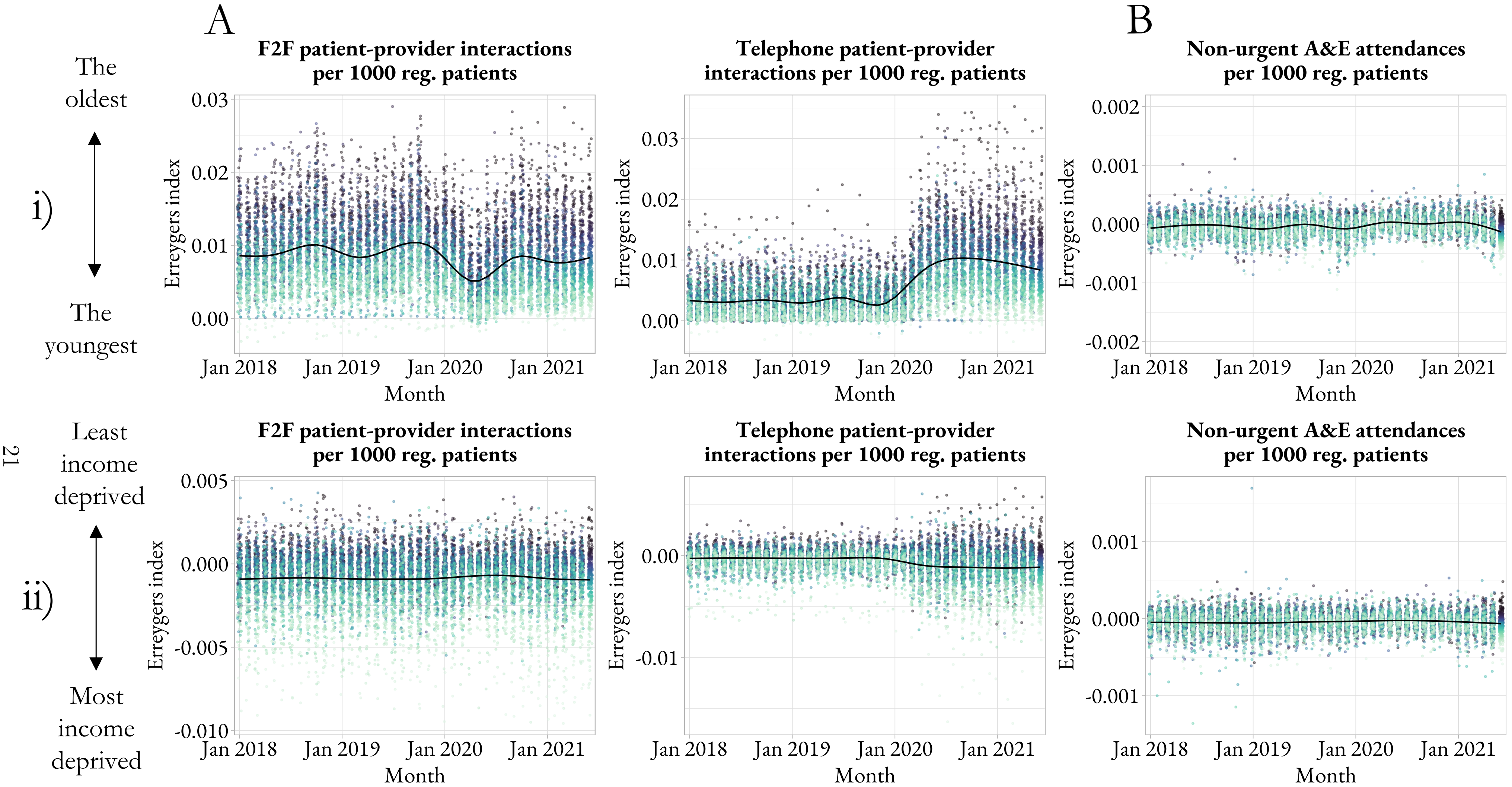

Supplementary Figure S5: Inequality in healthcare utilisation based on the Erreygers index. *Note:* A&E indicates Accident & Emergency department; F2F, Face-to-Face; reg., registered. Panels *i)* and *ii)* represent age and socioeconomic-related inequality, respectively. Panel A shows inequality in synchronous interactions in primary care and Panel B depicts inequality in non-urgent non-urgent A&E attendances. The data are between January 2018 and June 2021. Socioeconomic status is based on income-related deprivation levels. Each dot is a practice; practices are coloured according to their position in each outcome distribution as of June 2021, from light green (practices with lowest values) to dark grey (practices with highest values). (Continued)

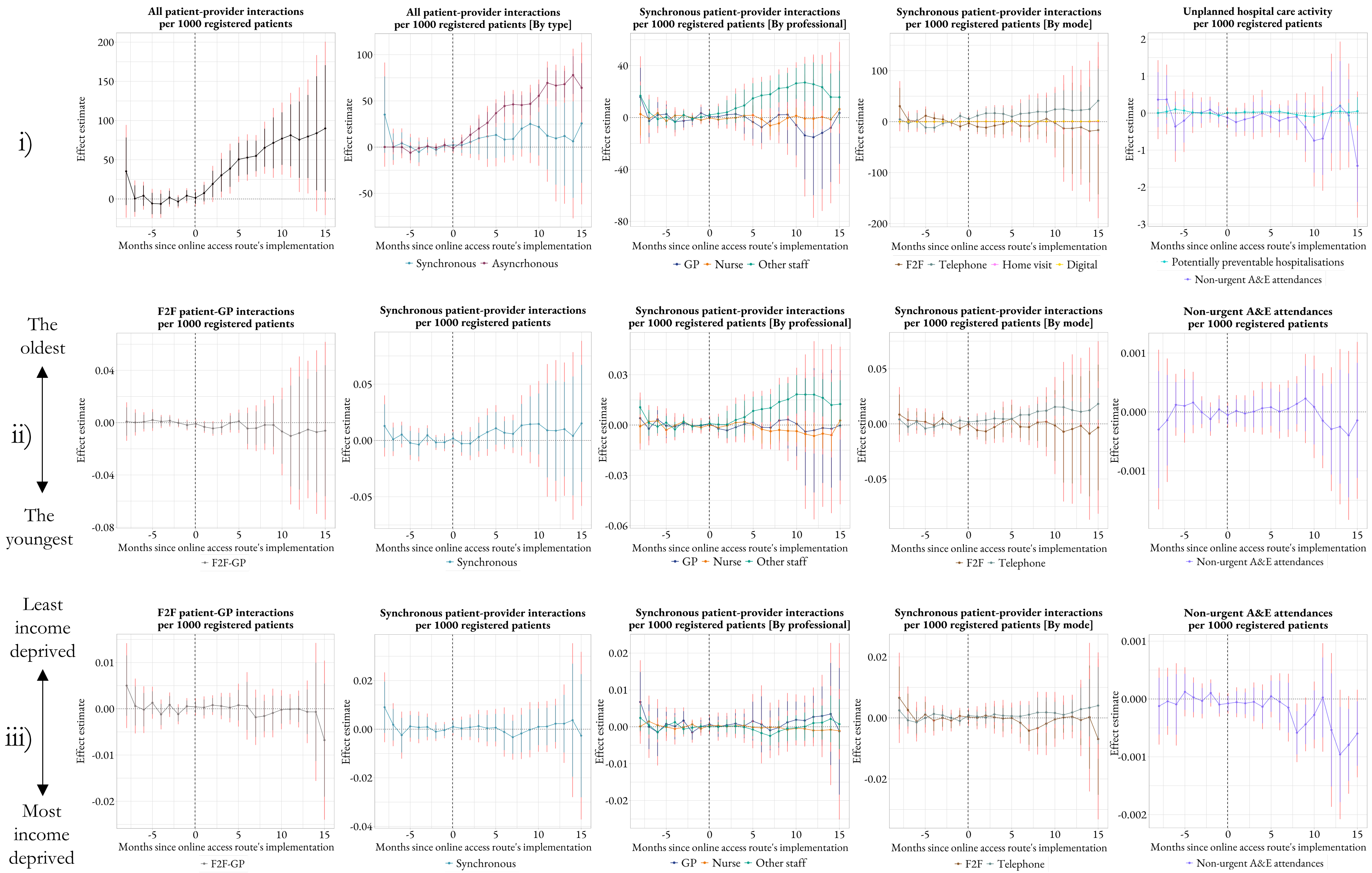

Supplementary Figure S6: Estimates of the average treatment effect on the treated at different months since implementation of the online access route to primary care from the unadjusted analysis (C) in 2020-21. *Note:* Panels *i)*, *ii)* and *iii)* show, respectively, the level, age and socioeconomic-related inequality in healthcare utilisation based on the generalised concentration index. Confidence intervals that account for potential issues arising from multiple testing are shown in red.

Supplementary Table S3: Analysis of potential treatment effect heterogeneity on the level of all patient-provider interactions according to practice's characteristics

Practice's characteristic	Estimate (95 % Confidence Interval)
**Average number of registered patients**	
Subsample of practices equal or above the median of the entire sample as of January 2019	76.076* (33.877, 118.275)
Subsample of practices below the median of the entire sample as of January 2019	120.524* (75.200, 165.849)
**Number of registered patients per full-time equivalent General Practitioner**	
Subsample of practices equal or above the median of the entire sample as of January 2019	92.989* (41.543, 144.435)
Subsample of practices below the median of the entire sample as of January 2019	125.545* (67.675, 183.416)
**Number of registered patients per full-time equivalent administrative/non-clinical**	
Subsample of practices equal or above the median of the entire sample as of January 2019	137.074* (106.749, 167.400)
Subsample of practices below the median of the entire sample as of January 2019	96.995* (17.758, 176.232)
**% of qualified and permanent General Practitioners who are female**	
Subsample of practices equal or above the median of the entire sample as of January 2019	105.571* (57.588, 153.554)
Subsample of practices below the median of the entire sample as of January 2019	91.400* (53.807, 128.993)
**% of qualified and permanent General Practitioners who are 55+ years**	
Subsample of practices equal or above the median of the entire sample as of January 2019	129.778* (70.959, 188.597)
Subsample of practices below the median of the entire sample as of January 2019	76.684* (35.190, 118.178)
**Average download speed (Mbit/s) at the practice**	
Subsample of practices equal or above the median of the entire sample as of January 2019	107.573* (45.720, 169.426)
Subsample of practices below the median of the entire sample as of January 2019	109.543* (71.271, 147.816)
**Average download speed (Mbit/s) at the residential area of registered patients**	
Subsample of practices equal or above the median of the entire sample as of January 2019	92.709* (31.546, 153.872)
Subsample of practices below the median of the entire sample as of January 2019	119.593* (80.173, 159.013)
**% of registered patients who reported to be easy† to contact the practice by phone**	
Subsample of practices equal or above the median of the entire sample as of January 2019	78.744* (22.855, 134.633)
Subsample of practices below the median of the entire sample as of January 2019	106.602* (53.613, 159.591)
**% of registered patients who reported receptionists to be helpful†**	
Subsample of practices equal or above the median of the entire sample as of January 2019	109.118* (54.384, 163.852)
Subsample of practices below the median of the entire sample as of January 2019	116.549 (67.418, 165.680)
**% of registered patients who reported to be easy† to use the practice's website**	
Subsample of practices equal or above the median of the entire sample as of January 2019	57.322* (7.430, 107.214)

Table S3 Analysis of potential treatment effect heterogeneity on the level of all patient-provider interactions according to practice's characteristics (Continued)

**Practice's characteristic**	**Estimate (95 % Confidence Interval)**
Subsample of practices below the median of the entire sample as of January 2019	164.194* (123.628, 204.761)
**% of registered patients who reported to be satisfied† with practice's appointment times**	
Subsample of practices equal or above the median of the entire sample as of January 2019	76.093* (31.626, 120.560)
Subsample of practices below the median of the entire sample as of January 2019	117.410* (71.479, 163.341)

Note:

* P-value < 0.05

† includes "fairly" or "very" as per Ipsos General Practice Patient Survey (https://gp-patient.co.uk)

Supplementary Table S4: Analysis of potential treatment effect heterogeneity on the level of asynchronous patient-provider interactions according to practice's characteristics

**Practice's characteristic**	**Estimate (95 % Confidence Interval)**
**Average number of registered patients**	
Subsample of practices equal or above the median of the entire sample as of January 2019	99.219* (64.043, 134.395)
Subsample of practices below the median of the entire sample as of January 2019	108.172* (71.322, 145.022)
**Number of registered patients per full-time equivalent General Practitioner**	
Subsample of practices equal or above the median of the entire sample as of January 2019	114.935* (92.534, 137.335)
Subsample of practices below the median of the entire sample as of January 2019	101.752* (61.229, 142.275)
**Number of registered patients per full-time equivalent administrative/non-clinical**	
Subsample of practices equal or above the median of the entire sample as of January 2019	109.809* (88.017, 131.602)
Subsample of practices below the median of the entire sample as of January 2019	96.783* (38.138, 155.427)
**% of qualified and permanent General Practitioners who are female**	
Subsample of practices equal or above the median of the entire sample as of January 2019	96.067* (62.188, 129.946)
Subsample of practices below the median of the entire sample as of January 2019	128.889* (104.006, 153.772)
**% of qualified and permanent General Practitioners who are 55+ years**	
Subsample of practices equal or above the median of the entire sample as of January 2019	108.995* (64.654, 153.335)
Subsample of practices below the median of the entire sample as of January 2019	100.371* (67.162, 133.581)
**Average download speed (Mbit/s) at the practice**	
Subsample of practices equal or above the median of the entire sample as of January 2019	98.582* (57.757, 139.406)
Subsample of practices below the median of the entire sample as of January 2019	116.031* (94.033, 138.030)
**Average download speed (Mbit/s) at the residential area of registered patients**	
Subsample of practices equal or above the median of the entire sample as of January 2019	82.041* (40.381, 123.701)
Subsample of practices below the median of the entire sample as of January 2019	127.921* (111.082, 144.761)
**% of registered patients who reported to be easy† to contact the practice by phone**	
Subsample of practices equal or above the median of the entire sample as of January 2019	72.833* (25.892, 119.773)
Subsample of practices below the median of the entire sample as of January 2019	114.913* (96.323, 133.502)
**% of registered patients who reported receptionists to be helpful†**	
Subsample of practices equal or above the median of the entire sample as of January 2019	74.758* (39.070, 110.445)
Subsample of practices below the median of the entire sample as of January 2019	125.661* (105.333, 145.989)
**% of registered patients who reported to be easy† to use the practice's website**	
Subsample of practices equal or above the median of the entire sample as of January 2019	65.405* (34.826, 95.984)

Table S4 Analysis of potential treatment effect heterogeneity on the level of asynchronous patient-provider interactions according to practice's characteristics (Continued)

**Practice's characteristic**	**Estimate (95 % Confidence Interval)**
Subsample of practices below the median of the entire sample as of January 2019	129.161* (110.179, 148.142)
**% of registered patients who reported to be satisfied† with practice's appointment times**	
Subsample of practices equal or above the median of the entire sample as of January 2019	66.702* (36.668, 96.735)
Subsample of practices below the median of the entire sample as of January 2019	121.063* (103.721, 138.405)

Note:

* P-value < 0.05

† includes "fairly" or "very" as per Ipsos General Practice Patient Survey (https://gp-patient.co.uk)

Supplementary Table S5: Analysis of potential treatment effect heterogeneity on the level of synchronous patient-provider interactions according to practice's characteristics

Practice's characteristic	Estimate (95 % Confidence Interval)
**Average number of registered patients**	
Subsample of practices equal or above the median of the entire sample as of January 2019	-21.545 (-51.375, 8.285)
Subsample of practices below the median of the entire sample as of January 2019	12.321 (-7.012, 31.654)
**Number of registered patients per full-time equivalent General Practitioner**	
Subsample of practices equal or above the median of the entire sample as of January 2019	-19.190 (-63.922, 25.542)
Subsample of practices below the median of the entire sample as of January 2019	23.502 (-9.954, 56.957)
**Number of registered patients per full-time equivalent administrative/non-clinical**	
Subsample of practices equal or above the median of the entire sample as of January 2019	29.845 (-6.040, 65.731)
Subsample of practices below the median of the entire sample as of January 2019	-0.907 (-29.516, 27.702)
**% of qualified and permanent General Practitioners who are female**	
Subsample of practices equal or above the median of the entire sample as of January 2019	9.552 (-25.651, 44.755)
Subsample of practices below the median of the entire sample as of January 2019	-32.574 (-78.247, 13.098)
**% of qualified and permanent General Practitioners who are 55+ years**	
Subsample of practices equal or above the median of the entire sample as of January 2019	19.971 (-12.800, 52.742)
Subsample of practices below the median of the entire sample as of January 2019	-20.232 (-71.909, 31.445)
**Average download speed (Mbit/s) at the practice**	
Subsample of practices equal or above the median of the entire sample as of January 2019	9.025 (-25.486, 43.536)
Subsample of practices below the median of the entire sample as of January 2019	-3.408 (-47.283, 40.468)
**Average download speed (Mbit/s) at the residential area of registered patients**	
Subsample of practices equal or above the median of the entire sample as of January 2019	11.189 (-23.946, 46.324)
Subsample of practices below the median of the entire sample as of January 2019	-7.126 (-40.598, 26.347)
**% of registered patients who reported to be easy† to contact the practice by phone**	
Subsample of practices equal or above the median of the entire sample as of January 2019	6.025 (-18.033, 30.083)
Subsample of practices below the median of the entire sample as of January 2019	-6.807 (-48.198, 34.584)
**% of registered patients who reported receptionists to be helpful†**	
Subsample of practices equal or above the median of the entire sample as of January 2019	34.773* (2.403, 67.143)
Subsample of practices below the median of the entire sample as of January 2019	-7.494 (-49.784, 34.796)
**% of registered patients who reported to be easy† to use the practice's website**	
Subsample of practices equal or above the median of the entire sample as of January 2019	-7.762 (-37.029, 21.506)

Table S5 Analysis of potential treatment effect heterogeneity on the level of synchronous patient-provider interactions according to practice's characteristics (Continued)

**Practice's characteristic**	**Estimate (95 % Confidence Interval)**
Subsample of practices below the median of the entire sample as of January 2019	36.393 (-0.187, 72.974)
**% of registered patients who reported to be satisfied† with practice's appointment times**	
Subsample of practices equal or above the median of the entire sample as of January 2019	9.695 (-22.873, 42.262)
Subsample of practices below the median of the entire sample as of January 2019	-2.140 (-48.219, 43.938)

Note:

* P-value < 0.05

† includes "fairly" or "very" as per Ipsos General Practice Patient Survey (https://gp-patient.co.uk)

Supplementary Table S6: Analysis of potential treatment effect heterogeneity on the age-related inequality in synchronous patient-provider interactions according to practice's characteristics

**Practice's characteristic**	**Estimate (95 % Confidence Interval)**
**Average number of registered patients**	
Subsample of practices equal or above the median of the entire sample as of January 2019	-0.010 (-0.032, 0.011)
Subsample of practices below the median of the entire sample as of January 2019	0.015 (-0.007, 0.037)
**Number of registered patients per full-time equivalent General Practitioner**	
Subsample of practices equal or above the median of the entire sample as of January 2019	-0.008 (-0.043, 0.027)
Subsample of practices below the median of the entire sample as of January 2019	0.026 (-0.006, 0.057)
**Number of registered patients per full-time equivalent administrative/non-clinical**	
Subsample of practices equal or above the median of the entire sample as of January 2019	0.025 (-0.007, 0.056)
Subsample of practices below the median of the entire sample as of January 2019	0.012 (-0.013, 0.037)
**% of qualified and permanent General Practitioners who are female**	
Subsample of practices equal or above the median of the entire sample as of January 2019	0.010 (-0.011, 0.030)
Subsample of practices below the median of the entire sample as of January 2019	-0.007 (-0.041, 0.027)
**% of qualified and permanent General Practitioners who are 55+ years**	
Subsample of practices equal or above the median of the entire sample as of January 2019	0.026 (-0.003, 0.055)
Subsample of practices below the median of the entire sample as of January 2019	-0.011 (-0.042, 0.020)
**Average download speed (Mbit/s) at the practice**	
Subsample of practices equal or above the median of the entire sample as of January 2019	0.011 (-0.019, 0.040)
Subsample of practices below the median of the entire sample as of January 2019	0.012 (-0.022, 0.047)
**Average download speed (Mbit/s) at the residential area of registered patients**	
Subsample of practices equal or above the median of the entire sample as of January 2019	0.012 (-0.015, 0.039)
Subsample of practices below the median of the entire sample as of January 2019	0.004 (-0.016, 0.024)
**% of registered patients who reported to be easy† to contact the practice by phone**	
Subsample of practices equal or above the median of the entire sample as of January 2019	0.010 (-0.013, 0.034)
Subsample of practices below the median of the entire sample as of January 2019	-0.002 (-0.031, 0.027)
**% of registered patients who reported receptionists to be helpful†**	
Subsample of practices equal or above the median of the entire sample as of January 2019	0.033* (0.003, 0.063)
Subsample of practices below the median of the entire sample as of January 2019	0.000 (-0.028, 0.027)
**% of registered patients who reported to be easy† to use the practice's website**	
Subsample of practices equal or above the median of the entire sample as of January 2019	0.000 (-0.027, 0.026)

Table S6 Analysis of potential treatment effect heterogeneity on the age-related inequality in synchronous patient-provider interactions according to practice's characteristics (Continued)

**Practice's characteristic**	**Estimate (95 % Confidence Interval)**
Subsample of practices below the median of the entire sample as of January 2019	0.032 (-0.009, 0.073)
**% of registered patients who reported to be satisfied† with practice's appointment times**	
Subsample of practices equal or above the median of the entire sample as of January 2019	0.003 (-0.025, 0.032)
Subsample of practices below the median of the entire sample as of January 2019	0.009 (-0.024, 0.042)

Note:

* P-value < 0.05

† includes "fairly" or "very" as per Ipsos General Practice Patient Survey (https://gp-patient.co.uk)

Supplementary Table S7: Analysis of potential treatment effect heterogeneity on the (income) deprivation-related inequality in synchronous patient-provider interactions according to practice's characteristics

Practice's characteristic	Estimate (95 % Confidence Interval)
**Average number of registered patients**	
Subsample of practices equal or above the median of the entire sample as of January 2019	-0.008* (-0.011, -0.005)
Subsample of practices below the median of the entire sample as of January 2019	0.000 (-0.006, 0.007)
**Number of registered patients per full-time equivalent General Practitioner**	
Subsample of practices equal or above the median of the entire sample as of January 2019	-0.002 (-0.010, 0.007)
Subsample of practices below the median of the entire sample as of January 2019	0.000 (-0.009, 0.009)
**Number of registered patients per full-time equivalent administrative/non-clinical**	
Subsample of practices equal or above the median of the entire sample as of January 2019	0.006 (-0.0003, 0.012)
Subsample of practices below the median of the entire sample as of January 2019	-0.003 (-0.007, 0.002)
**% of qualified and permanent General Practitioners who are female**	
Subsample of practices equal or above the median of the entire sample as of January 2019	-0.003 (-0.009, 0.003)
Subsample of practices below the median of the entire sample as of January 2019	0.003 (-0.005, 0.012)
**% of qualified and permanent General Practitioners who are 55+ years**	
Subsample of practices equal or above the median of the entire sample as of January 2019	0.004 (-0.005, 0.012)
Subsample of practices below the median of the entire sample as of January 2019	-0.006 (-0.014, 0.003)
**Average download speed (Mbit/s) at the practice**	
Subsample of practices equal or above the median of the entire sample as of January 2019	0.000 (-0.007, 0.006)
Subsample of practices below the median of the entire sample as of January 2019	0.002 (-0.004, 0.008)
**Average download speed (Mbit/s) at the residential area of registered patients**	
Subsample of practices equal or above the median of the entire sample as of January 2019	0.002 (-0.006, 0.009)
Subsample of practices below the median of the entire sample as of January 2019	-0.005 (-0.011, 0.0008)
**% of registered patients who reported to be easy† to contact the practice by phone**	
Subsample of practices equal or above the median of the entire sample as of January 2019	0.000 (-0.005, 0.006)
Subsample of practices below the median of the entire sample as of January 2019	-0.005 (-0.012, 0.001)
**% of registered patients who reported receptionists to be helpful†**	
Subsample of practices equal or above the median of the entire sample as of January 2019	0.002 (-0.003, 0.007)
Subsample of practices below the median of the entire sample as of January 2019	-0.001 (-0.007, 0.004)
**% of registered patients who reported to be easy† to use the practice's website**	
Subsample of practices equal or above the median of the entire sample as of January 2019	-0.002 (-0.009, 0.004)

Table S7 Analysis of potential treatment effect heterogeneity on the (income) deprivation-related inequality in synchronous patient-provider interactions according to practice's characteristics (Continued)

**Practice's characteristic**	**Estimate (95 % Confidence Interval)**
Subsample of practices below the median of the entire sample as of January 2019	0.005 (-0.0004, 0.010)
**% of registered patients who reported to be satisfied† with practice's appointment times**	
Subsample of practices equal or above the median of the entire sample as of January 2019	-0.002 (-0.009, 0.004)
Subsample of practices below the median of the entire sample as of January 2019	-0.001 (-0.010, 0.008)

Note:

* P-value < 0.05

† includes "fairly" or "very" as per Ipsos General Practice Patient Survey (https://gp-patient.co.uk)

Supplementary Table S8: Analysis of potential treatment effect heterogeneity on the level of synchronous patient-provider interactions according to characteristics of the registered patient population

Patient population's characteristic	Estimate (95 % Confidence Interval)
**Average age of registered patients**	
Subsample of practices equal or above the median of the entire sample as of January 2019	17.72 (-12.89, 48.32)
Subsample of practices below the median of the entire sample as of January 2019	7.53 (-18.04, 33.11)
**Average income decile of registered patients**	
Subsample of practices equal or above the median of the entire sample as of January 2019	6.70 (-31.10, 44.50)
Subsample of practices below the median of the entire sample as of January 2019	3.29 (-41.28, 47.86)
**% of registered patients who are female**	
Subsample of practices equal or above the median of the entire sample as of January 2019	3.94 (-36.33, 44.21)
Subsample of practices below the median of the entire sample as of January 2019	7.75 (-31.18, 46.67)

Note:

* P-value < 0.05
